# Nocturnal surveys of lined seahorses reveal increased densities and seasonal recruitment patterns

**DOI:** 10.1002/ece3.9573

**Published:** 2023-01-11

**Authors:** Heather D. Mason, Emily Rose, Jessica Elson Gonzalez, Duncan A. O'Brien

**Affiliations:** ^1^ The University of Tampa Tampa Florida USA; ^2^ The Center for Ocean Research and Education Gregorytown, Eleuthera The Bahamas; ^3^ Present address: Department of Biology Valdosta State University Valdosta Georgia USA; ^4^ Present address: School of Biological Sciences University of Bristol Bristol UK

**Keywords:** behavior, cathemeral, conservation, *Hippocampus erectu*s, nocturnal, recruitment

## Abstract

Although the nighttime ecology of organisms remains understudied, nocturnal surveys play an integral part in assessing fish assemblages and the selective forces shaping them. Eleuthera (Bahamas) contains an unusual population of lined seahorses (*Hippocampus erectus*) in an anchialine lake, possessing morphological characteristics distinct from those found in the ocean. Population surveys for seahorses and their potential predators were conducted at midnight and midday during wet and dry seasons, with belt transects perpendicular to the shoreline that increased in depth away from shore. Nocturnal surveys uncovered seahorse densities 259% higher than daytime transects on average. Sex ratios were consistently male‐biased, and the frequency of animals from different reproductive categories varied significantly by time of day, with gravid males observed around the clock but females and nongravid males observed more often at night. Spatial and seasonal recruitment was detected for the first time in this species, with an increase in juveniles detected in the shallow ends of transects during dry season surveys. Juvenile recruitment is poorly understood across syngnathid fishes, so the detection of early recruits at night has broad implications for this fish family. Seahorses from all reproductive categories were perched significantly higher in the water column during the night regardless of their depth or season. Predator densities followed a similar pattern with higher densities observed at night, indicating that elevated nocturnal perch height may be a response to predator presence. However, the selective agents driving these nocturnal behaviors have yet to be identified. Considering *H. erectus* is listed on the IUCN Red List as “Vulnerable,” the increase in nocturnal population size and the detection of juveniles has crucial implications for understanding their ecology, recruitment, and conservation.

## Graphical abstract

Night reveals lined seahorses from an isolated saltwater lake displaying behaviors previously undescribed when compared to daytime. Far more seahorses were observed at night compared with day, with evidence of nocturnal juvenile recruitment. We observed cryptic behaviors during the day and upright body posture and higher position in the water column at night. We investigated predators that may drive these behaviors, as potential predator density was also higher at night than during the day.

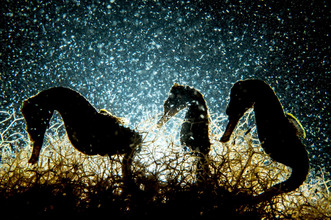

## 1 INTRODUCTION

As a diurnal species, humans have strongly biased our understanding of the population dynamics and behavioral patterns of many species by solely studying them during the day, which can have critical implications for their management in the wild. Until recently, we knew little about the biology of organisms at night, but with the advent of low‐light trap cameras and other technology, specific behaviors, their selective factors, and the nocturnal activity cycle are coming into focus (Gaston, 2019). “Night” poses vastly different selective pressures across environments, which extend well beyond the simple lack of light (Gaynor et al., 2018). In shallow water systems, air and water temperatures fluctuate between day and night, leading to changes in thermoclines, wind, and water circulation patterns. The nocturnal changes in climate can affect dissolved oxygen levels, cause planktonic shifts or alter broader community dynamics, and shift overall ecosystem and organism functioning. Such organismal‐level effects have recently been exemplified by changes in gene activity under constant light versus intermittent light in Acroporid corals (Gaston, 2019; Rayner, 2003; Reeve, 1964; Reyes & Merino, 1991; Rosenberg et al., 2019; Sameoto, 1986).

Animals active at night display a range of unique behaviors associated with conducting the ordinary business of life in the absence of ambient light and a suite of behaviors specific to the nighttime hours (Nichols & Alexander, 2018). Most animals are clearly either nocturnal or diurnal, but in some groups, a range of activity cycles exist. Recent estimates suggest that 30% of vertebrates and 60% of invertebrates display nocturnality (Hölker et al., 2010). Alternatively, some animals are cathemeral, possessing flexible patterns of activity around the clock depending on food availability and season (Bennie et al., 2014; Colquhoun, 2006; Eppley & Donati, 2019). Others, like Merriam's kangaroo rats (*Dipodomys merriami*), are crepuscular and displayed their highest activity levels at twilight (Daly et al., 1992). Lunar cycles are similarly influential upon the activity levels of animals active at night, with some mammals displaying suppressed activity during the full moon (Prugh & Golden, 2014). Among invertebrates, some taxa show predominantly nocturnal activity (like fireflies; Lewis, 2016), with others displaying a range of activity cycles. Intra‐ and interspecific variation in peak activity can consequently be related to predation risk, resource availability, environmental conditions, ontogenetic differences, evolutionary history, and human influences among others (Bennie et al., 2014; Flecker, 1992; Gaynor et al., 2018; Hammerschlag et al., 2010; Nichols & Alexander, 2018; Prugh & Golden, 2014; Tagg et al., 2018).

Fish demonstrate a range of activity cycles, including fully diurnal, entirely nocturnal, crepuscular, or cathemeral (Aguzzi et al., 2013; Clark et al., 2009; Fox & Bellwood, 2011; Lin et al., 2013; Reebs, 1992). Even fish within the same species located in contiguous habitats can display differences; the golden‐lined rabbitfish (*Siganus lineatus*) displayed diurnal foraging along shorelines, but reef populations foraged most commonly at night (Fox & Bellwood, 2011). Predation risk specifically has been shown to influence diel activity in many fish species, including minnows, guppies, carp, bluestripe grunt, gray snapper, and sea breams among others (Fraser et al., 2004; Hammerschlag et al., 2010; Metcalfe & Steele, 2001; Pettersson et al., 2001; Reebs, 2002). However, temperature variation, ontogeny, prey availability, nutritional status, lunar phase, and reproductive status have also been shown to be critical to the level of nocturnal activity in fish (Clark et al., 2009; Fraser et al., 1993; Gries et al., 1997; Metcalfe & Steele, 2001; Nagelkerken et al., 2000; Reebs, 2002; Reebs et al., 1984). The collection of research supporting a wide range of behaviors in fish throughout the day indicates the potential for many species to display diel changes in activity that have yet to be observed.

To effectively assess the conservation status and overall ecological niche of a species, it is vital to know when individuals are most active or most easily censused. Differences in the detectability of organisms between night and day play a role in accurate measures of animals' populations, not always with the expected consequence that animals are more detectable during the day. In bull trout (*Salvelinus confluentus*) and smallmouth bass (*Micropterus dolomieu*), nighttime visual counts and electrofishing census data estimated higher population sizes than during the day (Blackwell et al., 2017; Thurow & Schill, 1996). Similarly, the use of camera traps confirms that the presence of humans can reduce animal activity and detectability in multiple species (Swann & Perkins, 2014), and thus, daytime counts will often be lower than nighttime counts because animals can see researchers from further away and react accordingly.

An additional challenge in the detection of animals involves the morphological or behavioral adaptations that facilitate blending in with the landscape. In highly cryptic species like seahorses, daytime sampling can further bias against detection, leading to inappropriate management decisions (Aylesworth et al., 2017). The majority of seahorse species surveyed have been identified as diurnal, with anecdotal observations of nocturnality in the Pacific seahorse (*Hippocampus ingens*; Foster & Vincent, 2004), pot‐bellied seahorse (*H. abdominalis*; Martin‐Smith & Vincent, 2005; Paulin, 1992), and tiger‐tail seahorse (*H. comes*; Foster & Vincent, 2004; Perante, 2002). Although tiger‐tail seahorses were never observed during the day across a 24‐month study, seahorses were most visible at night high on top of the reef substrate (Perante, 2002). Researchers hypothesized that nocturnality in *H. comes* resulted from intense fishing pressures stemming from the aquaria and traditional medicine trades (Foster & Vincent, 2004). However, nocturnality or increased detectability at night may be more broadly present in this genus. If nocturnal activity is more common in seahorses, population monitoring might require surveys at night to more accurately census members of this CITES Appendix II in Appendix S1 trade‐protected genus (https://www.cites.org/eng/app/appendices.php).

Previous research has established that the Sweetings Pond marine lake (Eleuthera, The Bahamas) supports a range‐restricted population of an IUCN Red List “Vulnerable” seahorse species (lined seahorse, *Hippocampus erectus*) of higher density than observed in nearly any other seahorse population globally (Correia et al., 2018; Masonjones et al., 2019; Masonjones & Rose, 2019; Rose et al., 2016). Surveys of this population have consistently indicated the presence of more males than females. This is unusual for seahorse populations, which typically display female‐biased or equal sex ratios associated with their monogamous mating system (Foster & Vincent, 2004; Masonjones & Rose, 2019). This high‐density, male‐biased population provides a unique system to assess interactions between the sexes and their behaviors over a 24‐h diel cycle. Previous work included formal population sampling during the day (Masonjones et al., 2019) with animals observed engaging in cryptic behaviors in groups fewer than four seahorses (animals camouflage and tuck themselves away within the structure of the habitat) consistent with the behavior of other seahorse species (Freret‐Meurer et al., 2017). Both formal nocturnal fish census using baited remote underwater video (BRUV) and informal nighttime observations in Sweetings Pond suggest that this population of *H. erectus* may be nocturnal, engaging in noncryptic behaviors, including congregating in large social groups of up to 14 animals, perching on the top of vegetation, and performing courtship displays (Mason, unpublished data; O'Brien et al., 2021).

This study provides the first comprehensive survey of wild seahorses at night, utilizing a paired transect design to document daytime and nighttime seahorse population counts. We investigated the selective factors that may influence their nocturnality by focusing on three main questions centered around the seahorses' behaviors and the presence of potential predators. The primary question centers on how population densities, sex ratios, and ontogenetic shifts in detectability or habitat usage, i.e., water depth and distance from shore, may vary between day and night. Secondly, we aimed to determine how seahorse behaviors might shift between midday and midnight, in terms of how high they are on vegetation in the water column (perch height), their overall body position, and holdfast preference. Finally, our study identifies the potential predator pool in this unique, closed system and how potential predator densities relate to seahorse abundance between daytime and nighttime surveys. Both octopus and crabs are occasional predators of seahorses in other systems, and we have witnessed (August 2017) one act of nocturnal predation of a male seahorse by a spider crab. As a result, we also document predator presence during our diurnal and nocturnal surveys to identify potential selective pressures acting on this population of seahorses that could cause the seahorses to shift their activity cycle from day to night.

## 2 MATERIALS AND METHODS

### 2.1 Experimental design

**FIGURE 1:**
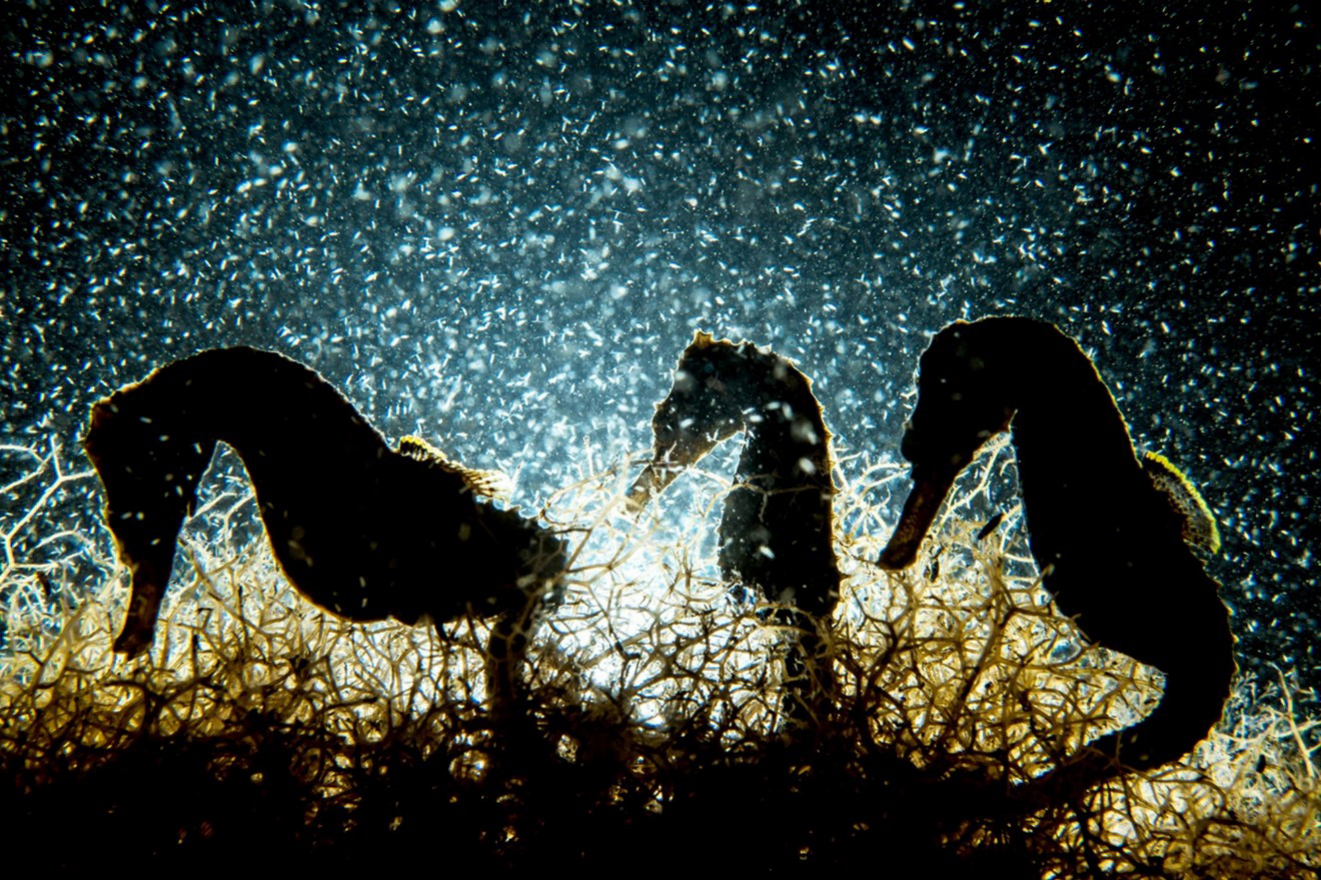
Image taken by Shane Gross of Sweetings Pond lined seahorses at night.

**FIGURE 2:**
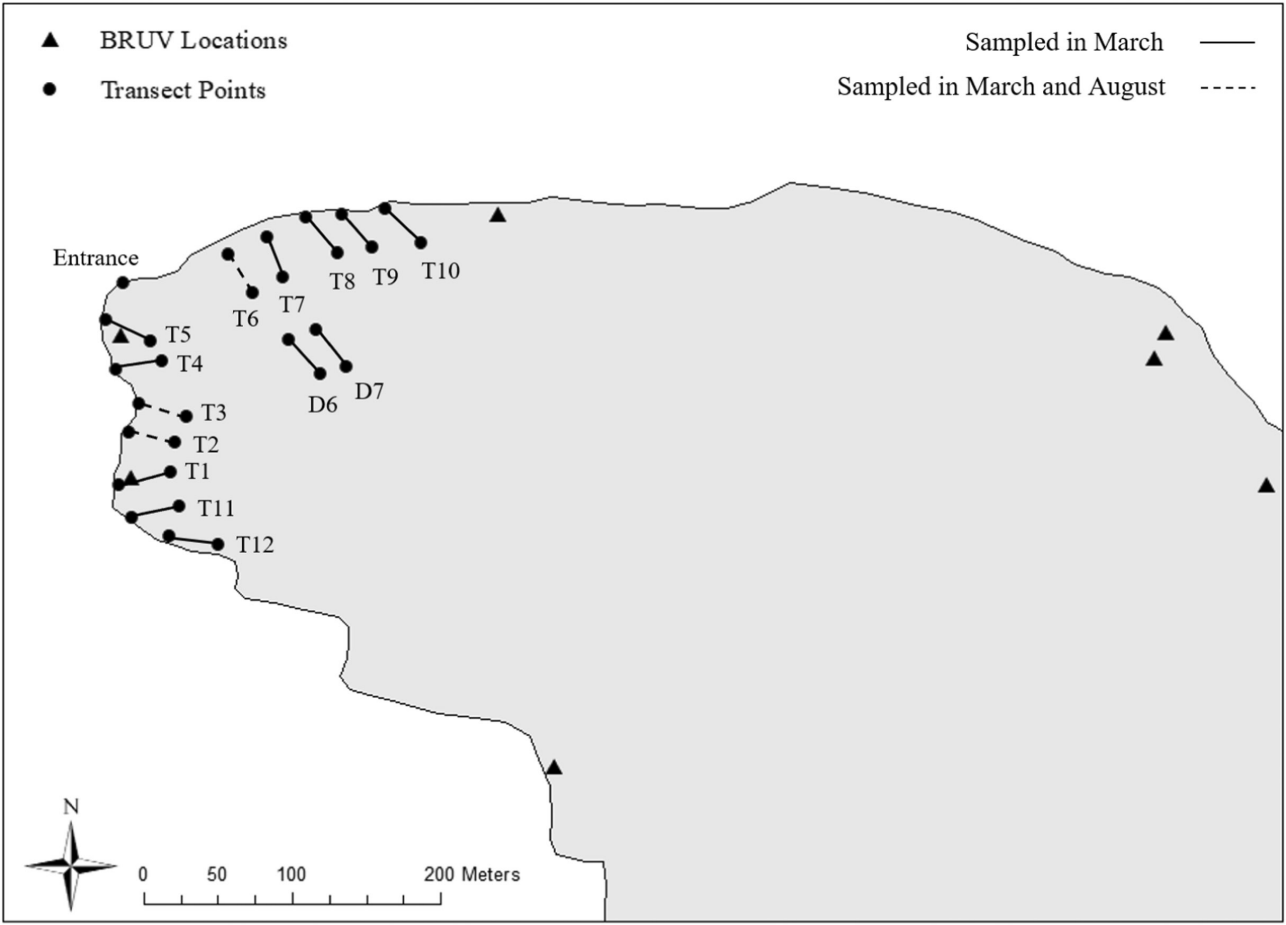
Location of research site, Sweetings Pond (N 25.21.40, W 076.30.40). Transects (30 by 2 M) are indicated by dots with lines, with solid lines sampled in march only and dashed lines sampled in March and August. Two deeper water transects were conducted, but not included in the general analysis, indicated as D transects in figure. Map projected in UTM Zone 18N, WGS84, meters.

The study organism was the lined seahorse, *Hippocampus erectus* (Figure 1), and the research site was a tidal saltwater lake located on the island of Eleuthera, The Bahamas (Figure 2; Masonjones et al., 2019). Field research was conducted with approval from the Bahamian government through the BEST Commission (Bahamas Environment, Scientific, Technology; separate approvals for 2014–2018). In addition, all animal use protocols described were approved under the University of Tampa Animal Use Protocol, AUP #2018‐1.

Day and night transects for *H. erectus* and their local predators were performed at the northwest end of the lake during the dry season (March 2018; *n* = 12) and the wet season (August 2018; resampled same transects, *n* = 3). This site was selected because of its consistently high daytime seahorse population across seasons and is identified by Masonjones et al. (2019) as the “Caves” site. Due to this being a high‐density site, we aimed to maximize coverage across the entire northern reach of the pond and consequently employed a systematic rather than random sampling approach. A 400‐meter section of shoreline was identified prior to sampling, with GPS points generated for 12 equidistant transects (average separating distance of 33 m; Figure 2). Transects were organized perpendicular to the shoreline, beginning in 1 m of water and GPS locations obtained for each end of the transect. Seahorse populations and their potential predator populations were surveyed on each 30 × 2 m belt transect between the hours of 10:00–14:00 (day) and 22:00–02:00 (night) in a paired sampling design. Transects were weighted and left in the field to enable repeat sampling at night. We used the three transects with the highest overall seahorse densities reported in the dry season (March 2018) to resample day and night in the wet season (August 2018) following the same protocol (Figure 2) to enable seasonal comparisons. We chose to resample the same locations because seahorses have been shown to display high site fidelity in other systems (Foster & Vincent, 2004). Two deeper water transects were sampled both day and night during March sampling (identified as D transects in Figure 2). Due to sampling time constraints, we were unable to replicate these deeper water transects more broadly and excluded them from the analysis.

### 2.2 Field methods

#### 2.2.1 Seahorse sampling

During daytime sampling, 1 m on each side of the tape was surveyed for seahorses, noting their depth in the water column, the location relative to the transect (in meters), side (right or left), and whether the animal was a juvenile, female, or male. Male reproductive status was identified in the field as either nongravid (not carrying embryos) or gravid (carrying embryos). In addition, we measured perch height as the distance from the sediment to the top of the seahorses' head in centimeters with a metric tape measure.

We photographed (using an Olympus TG‐5) animals in situ to identify holdfast species, with a closeup of the left side of the head of the animal taken if possible, to allow the identification of individuals. Divers did not have any physical contact with animals during the day to reduce the likelihood of relocation, which has been observed in a broad range of seahorse species after interactions with divers and their cameras (De Brauwer et al., 2018, 2019; Giglio et al., 2018; Harasti & Gladstone, 2013). During nighttime sampling, we took the same series of measurements for each animal and photographed them against a 1 cm grid background for size measurements.

#### 2.2.2 Potential predator sampling

To identify potential predators in the area surrounding each transect, we used two survey methods. The first included surveying the shorelines near the start of the transects during the daytime for spider crabs (*Maguimithrax spinosissimus*), Nassau grouper (*Epinephelus striatus*), and octopus (*Octopus briareus*) after we laid the transects initially. We investigated approximately 15 m of shoreline habitat on each side of the transect's starting point (average shoreline distance surveyed 24.4 (SD 1.4) m, total distance surveyed 381 m across 15 transects), including accessible cave structures, holes, and other underwater features, such as vegetation and sponges. During surveys, we scored underwater ledges as shallow (1 m or less), mid (1–2 m), or deep (greater than 2 m). For our second method of assessment, predators were documented during both daytime and nighttime surveys when observed within 2 m on each side of the transect tape (4 × 30 m total area sampled per transect). For both methods, predators were photographed with a scale in the image to obtain body size estimates, followed by calculating predator densities for each. We assessed what predators were eating through a visual scan of each one, identifying whether seahorses are a component of their diet.

#### 2.2.3 Habitat assessment

In order to assess the benthic habitat available for use in the system, photographs of the benthic cover were taken 1 m above the tape at each meter mark with 40 cm in each frame. To assess seahorse habitat preference, we compared habitat availability from benthic photos with in situ photos of seahorses on specific holdfasts both day and night.

### 2.3 Statistical analysis

#### 2.3.1 Population density

Statistical analyses were performed using JMP 11.2 (JMP, 2019). All means are provided with standard error in parentheses. We estimated total seahorse density as the total number of seahorses observed in a transect divided by transect area (60 m^2^). Densities and sex ratios were also estimated for the nearshore (the animals observed in first 0–15 m) and farshore sections (the deeper 15–30 m) of the transects to reflect patterns by depth and by distance to shoreline and thus potential predator pools. These densities were estimated by dividing seahorse counts by the area of a half transect (30 m^2^). Seahorse density did not have a normal distribution (Shapiro–Wilk, *W* = 0.867, *p* = .0008) and had unequal variances by the time of day (Levene's, *F*_1,31_ = 16.42, *p* < .0003) but not by season (Levene's, *F*_1,31_ = 0.27, *p* = .605). To determine differences in total seahorse density between night and day on each transect, we used a Wilcoxon Signed Rank test because the variables were not parametric. Secondly, repeated measures MANOVAs were used to determine the relationship between density at different times of day by season and by location on transects. Adult density followed the same distribution as total density, and thus the same statistical approach was used.

#### 2.3.2 Reproduction

We estimated the sex ratio as the total number of adult males divided by the total number of adults in the population. The sex ratio was normally distributed (Shapiro–Wilk, *W* = 0.95998, *p* = .2581) and displayed equal variances (Levene's test, *F*_1,31_ = 1.2032, *p* = .281, by season). We first used one‐sample *t*‐tests, with Bonferroni corrections applied for multiple tests following Holm (1979), to assess whether sex ratios deviated from a hypothesized 0.5 ratio. Paired *t*‐tests investigated differences in sex ratios on each transect between day and night. Next, we investigated seasonal differences in adult sex ratio with repeated measures MANOVA for changes in sex ratio by the time of day (continuous variable) and season (grouping variable). Counts of animals in four reproductive categories (juvenile, female, gravid, and nongravid male) were delineated for both half and full transects and investigated for patterns in frequency shifts between night and day and between the time of day and season using a contingency table likelihood‐ratio test.

#### 2.3.3 Body size

Image J (Rueden et al., 2017) was used to measure seahorse standard length (head, torso, and tail), following procedures in González et al., 2014; Rose et al., 2016. We confirmed seahorse sex, reproductive status (juvenile, female, male, gravid male), and holdfast habitat from photos. Because seahorses were not photographed on the grid backgrounds during the day in this study to prevent displacing them from their original location, dry season (March) and wet season (August) nocturnal size distributions of animals were compared with historic survey data. This data originated from Masonjones et al. (2019), where fish were photographed during March (2014–2016) and August (2015) on daytime transects in the “Caves” location, the same location as the current study. Size variables were not normally distributed (Shapiro–Wilk, *W* = 0.991, *p* < .0001) and had unequal variances (Levene's test, *F*_1,556_ = 56.2996, *p* < .0001). As a result, a general linear model (GLM) with a Poisson distribution was used to identify potential patterns in fish sizes with the time of day and season. Differences in the frequency distributions of body sizes were calculated using a series of two‐sample Kolmogorov–Smirnov tests. Differences in adult body sizes were explored by excluding all fish below 65 mm from analysis (the size at first reproduction in this population of *H. erectus*; Masonjones et al., 2019) before applying a GLM as the distribution was neither normal (Shapiro–Wilk, *W* = 0.940, *p* < .0001) nor had equal variances (Levene's test, *F*_1,735_ = 11.520, *p* = .0007).

#### 2.3.4 Habitat use and preference

To investigate seahorse body position relative to the substrate (*n* = 737 fish), we used in situ photos of seahorses on their holdfasts, categorizing them as 0 (laying flat on substrate or upside down, i.e., 90° to gravity), 0.5 (anywhere in between laying flat and vertically upwards, i.e., 90° to 180° tail to torso), and 1 (being completely upright relative to the substrate). Body posture was not normally distributed nor had equal variances and was investigated relative to the time of day and distance from shore using a general linear model. We used a Nominal Logistic Fit to investigate potential differences in habitat use to see the combined effects of sex and time of day on holdfast use.

Seahorse perch height was not normally distributed (Shapiro–Wilk, *W* = 0.9158, *p* < .0001) and had unequal variances (Levene's test, *F*_1,878_ = 25.163, *p* < .0001). Because of the variability in possible perch heights due to variable benthic cover on each specific transect, perch height for all fish was averaged per transect and then compared by time of day and by relation to shore and season using a GLM.

Coral Point Count (Kohler & Gill, 2006) was used to analyze the benthic cover's content at each meter along the 30‐m transects from benthic photos. We identified a 30 × 30 cm region from each photo, assigned 30 random points, and then the identity of the item under the point was classified to species level if possible. The percentage cover of each habitat type per meter on each transect was then estimated from these count data. We used the Manly‐Chesson Index to calculate seahorse holdfast preference, where *α* equals ∑i=1mripi, with *m* = number of benthic/holdfast categories used in the analysis, *r*_*i*_ = the proportion of seahorses on a particular holdfast in either the night or the day, and *p*_*i*_ = the proportion of that benthic type in the environment (Chesson, 1983; Manly et al., 1972). If *α* = 1/*m*, seahorses are using holdfasts relative to the frequency of that habitat component in the environment, but in cases where *α* < 1/*m* seahorses are avoiding that habitat component and *α* > 1/*m*, there is preferential usage.

#### 2.3.5 Potential predators

Differences between nighttime and daytime predator densities were investigated with a Wilcoxon Signed Rank test because daytime predator densities were not normal (Shapiro–Wilk, *W* = 0.557, *p* < .0001), with the relationship between predator density and both season and location on transect investigated with a repeated measures MANOVA. Finally, the relationship between the density of predators observed along the shoreline relative to the variables from perpendicular transects was estimated via linear regression.

## 3 RESULTS

### 3.1 Population density

During night surveys, seahorses were most commonly attached to the top of the vegetation, highly visible to divers, and were reported in numbers 376% higher during the night (*n* = 681) compared with the same transects during the day (*n* = 143) when counts are pooled. Consequently, nighttime lined seahorse densities were significantly higher independent of the season (Wilcoxon Signed Rank test, *S* = 68.0, *n* = 15, *p* < .0001), with nighttime densities on average 259.3 (32.5)% higher than daytime (Table 1). The highest density on a single nighttime transect was 1.67 seahorses m^−2^. Overall, there was no effect of season on total seahorse density (Repeated measures MANOVA, *F*_1,4_ = 2.39, *p* = .197) and no interaction effects (*F*_1,4_ = 6.972, *p* = .0576). Because there was no seasonal effect detected in total density (density of adults and juveniles combined), we combined March and August samples for any further statistical analyses.

**TABLE 1:** Table of variables related to *Hippocampus erectus* seahorse reproduction, provided as mean (SE) where appropriate

Variable	Day		Night	
Season	Dry season	Wet season	Dry season	Wet season
Total fish observed	100	43	582	99
Percent gravid males	59.0%	14.0%	30.6%	27.3%
Mean fish body length (mm; all fish)	86.55 (2.84)^a^	94.33 (1.10)^a^	80.51 (0.97)	91.07 (1.74)
Mean fish body length (mm; adults > 64 mm)	92.11 (2.44) (*n* = 48)^a^	94.94 (1.08) (*n* = 179)^a^	87.28 (0.92) (*n* = 425)	91.91 (1.68) (*n* = 85)
Number of juveniles (% of total fish observed)	12 (12.0%)	9 (20.9%)	128 (22.0%)	6 (6.1%)
Mean fish density (# m^−2^; *n* = 3 transects)	0.170 (0.02)	0.294 (0.01)	0.690 (0.10)	0.633 (0.23)
Mean adult fish density (# m^−2^; *n* = 3 transects)	0.18 (0.05)	0.24 (0.03)	0.90 (0.12)	0.60 (0.22)
Mean perch height (cm)	2.60 (0.49)		10.04 (0.66)	
Mean body position	0.141 (0.025)		0.606 (0.027)	

^a^ Daytime body lengths from Masonjones et al. (2019).

**FIGURE 3:**
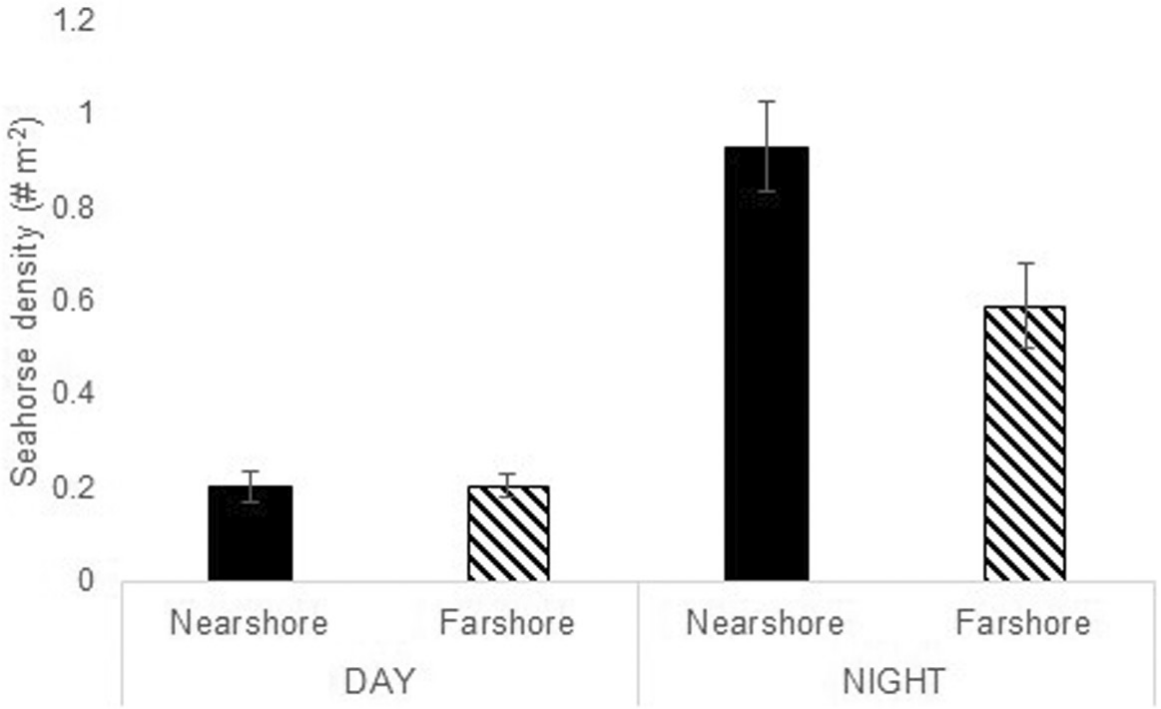
Mean total seahorse density (animals m^−2^, ±SE) compared between day and night and location relative to shore, with nearshore the first 15 × 2 m of transect and farshore the final 15 × 2 m of transect.

When investigated relative to distance from shore, overall seahorse density remained higher at night than during the day (Repeated measures MANOVA, *F*_1,28_ = 5.041, *p* = .0328; Figure 3). Seahorse density was higher closer to shore at night, a pattern not observed during the day (effect of the time of day: *F*_1,28_ = 74.071, *p* < .0001, distance to shore: *F*_1,28_ = 74.071, *p* < .0001, with significant interactions: *F*_1,28_ = 70.27, *p* = .013). To investigate the relationship between adult density and location on the transect, juveniles were excluded, which eliminated both the effect of distance from shore and season (Repeated measures MANOVA, distance: *F*_1,28_ = 2.299, *p* = .141; season: *F*_1,4_ = 0.716, *p* = .445), but the significantly higher nocturnal fish density was maintained (Wilcoxon Signed Ranks Test, *S* = 68.0, *n* = 15, *p* < .0001; Table 1).

### 3.2 Reproduction

**FIGURE 4:**
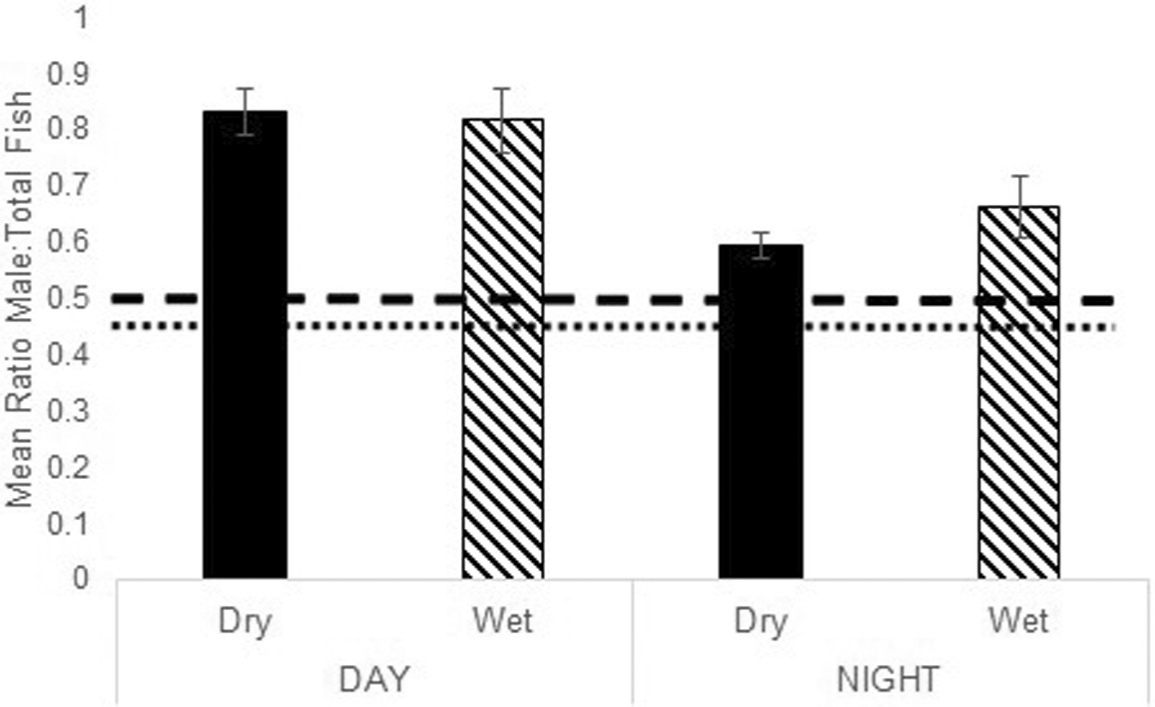
Ratio of males to total fish observed (±SE; sex ratio), with expectation given wild fish surveys of a ratio of 0.5 (dashed line). The dotted line indicates the mean seahorse sex ratio calculated across all seahorse species studies from Masonjones and Rose (2019).

We investigated variation in sex ratio across time of day and season in two ways. First, we determined whether this population deviated from an even (0.5) sex ratio typical for monogamous species by time of day. Overall, both day and night sex ratios deviated from 0.5, indicating that transects were significantly male‐biased throughout the entire study (Day X̅(SE) = 0.79 (0.04), *n* = 16, *t* = 6.651, df = 15, *p* < .0001; Night X̅(SE) = 0.60 (0.02), *n* = 16, *t* = 4.467, df = 15, *p* = .0005, adjusted *α* = .025; Bonferroni adjustment, Holm, 1979). Second, we compared the sex ratio between treatments to investigate the effect of time of day and season. Sex ratio was significantly influenced by time of day, with higher sex ratios (male‐biased) during the daytime compared with nighttime (Figure 4; paired *T*‐test, *t* = −3.712, df = 15, *p* = .002). In the subset of transects observed in both March and August, there was no effect of season on observed sex ratios (Repeated Measures MANOVA, *F*_1,4_ = 0.372, *p* = .575) with no interaction effects detected (*F*_1,4_ = 0.613, *p* = .477). In addition, sex ratio patterns with the time of day did not differ with distance to shore (Repeated measures MANOVA, *F*_1,28_ = 0.0581, *p* = .811).

**FIGURE 5:**
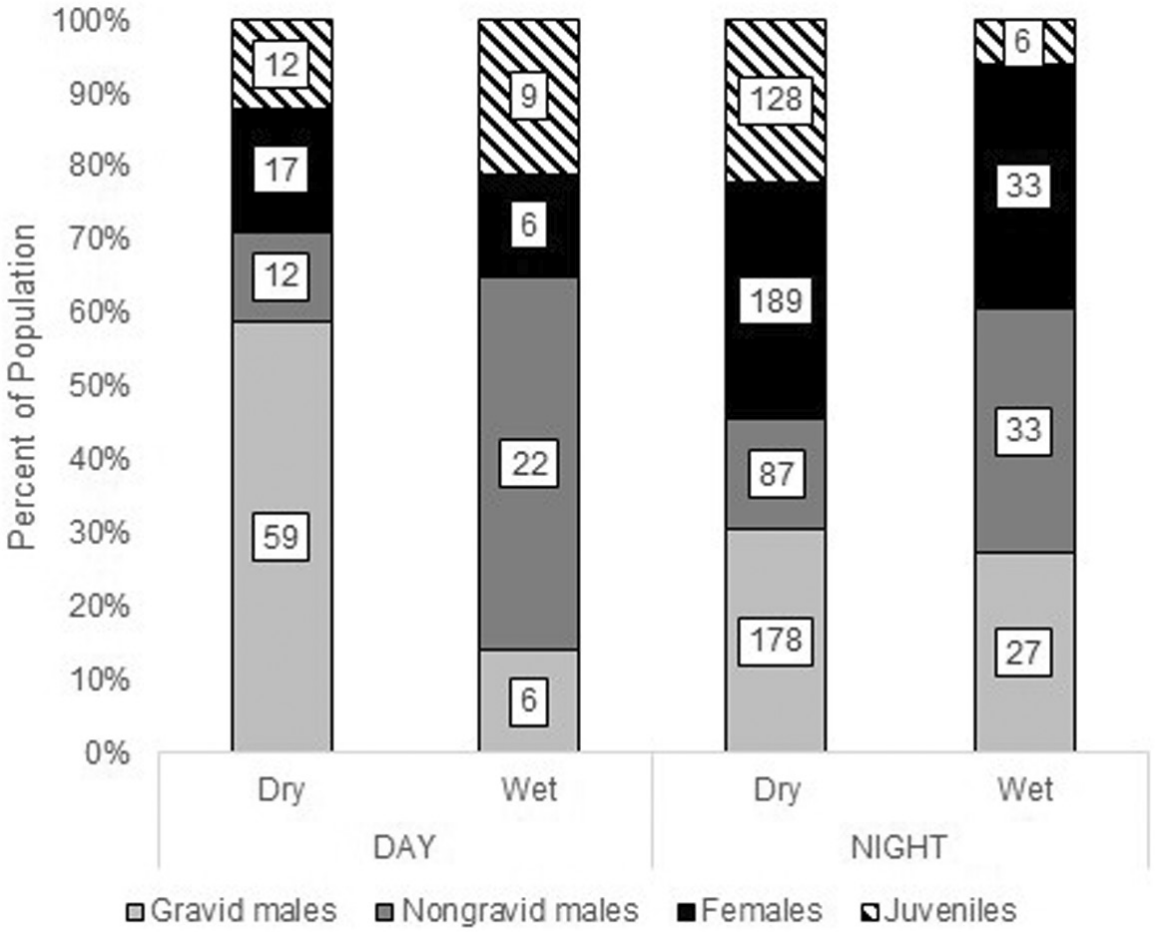
Frequency of reproductive categories by time of day (day/night) and season (wet/dry). Males in gray‐shaded bar sections, females in black bar sections, and juveniles in hatched bar sections. The number of individuals in each category indicated in each bar section.

Identifying the frequency of seahorses at different reproductive stages can provide insights into their population structure. Strong diel differences existed in the frequency of female, nongravid male, gravid male, and juvenile seahorses but only during the dry season (Contingency Table Analysis, *X* ^2^ = 30.133, df = 3, *p* < .0001; Figure 5). Diurnally, females, juveniles, and nongravid males each represented only 1.8%–2.5% of the total number of fish observed in the dry season, compared with 13%–28% of the population at night. Gravid males were more consistently observed, although fewer were observed overall during the day compared with at night (8.7% of the population during the day and 26% of the population at night).

Juvenile abundance showed the strongest statistical association with season, with 82% of all juvenile seahorses observed in the study seen at night during the dry season (Contingency Table Analysis, *X* ^2^ = 20.194, df = 1, *p* < .0001; Figure 5). Overall, males differed seasonally and by periods, with 62.5% of all males observed by this study surveyed at night in the dry season (Contingency Table Analysis, *X* ^2^ = 4.226, df = 1, *p* = .040). Nongravid males were equally common day and night in the wet season but were much more common at night during the dry season (Contingency Table Analysis, *X* ^2^ = 15.432, df = 1, *p* < .0001). For each time of day, there was no effect of season on the frequency of females in the population; they were generally uncommon during the day and more common at night (Contingency Table Analysis, *X* ^2^ = 1.732, df = 1, *p* = .188). Gravid males comprised 18%–25% of the population during the day, and 75%–82% of the population at night, with no differences by season (Contingency Table Analysis, *X* ^2^ = 0.754, df = 1, *p* = .3852). Although this appears contrary, what shifts is the abundance of nongravid males, with many more during the wet season and many fewer overall during the dry season; thus there is a higher ratio of gravid to nongravid males in the dry season than in the wet.

### 3.3 Body size

**FIGURE 6:**
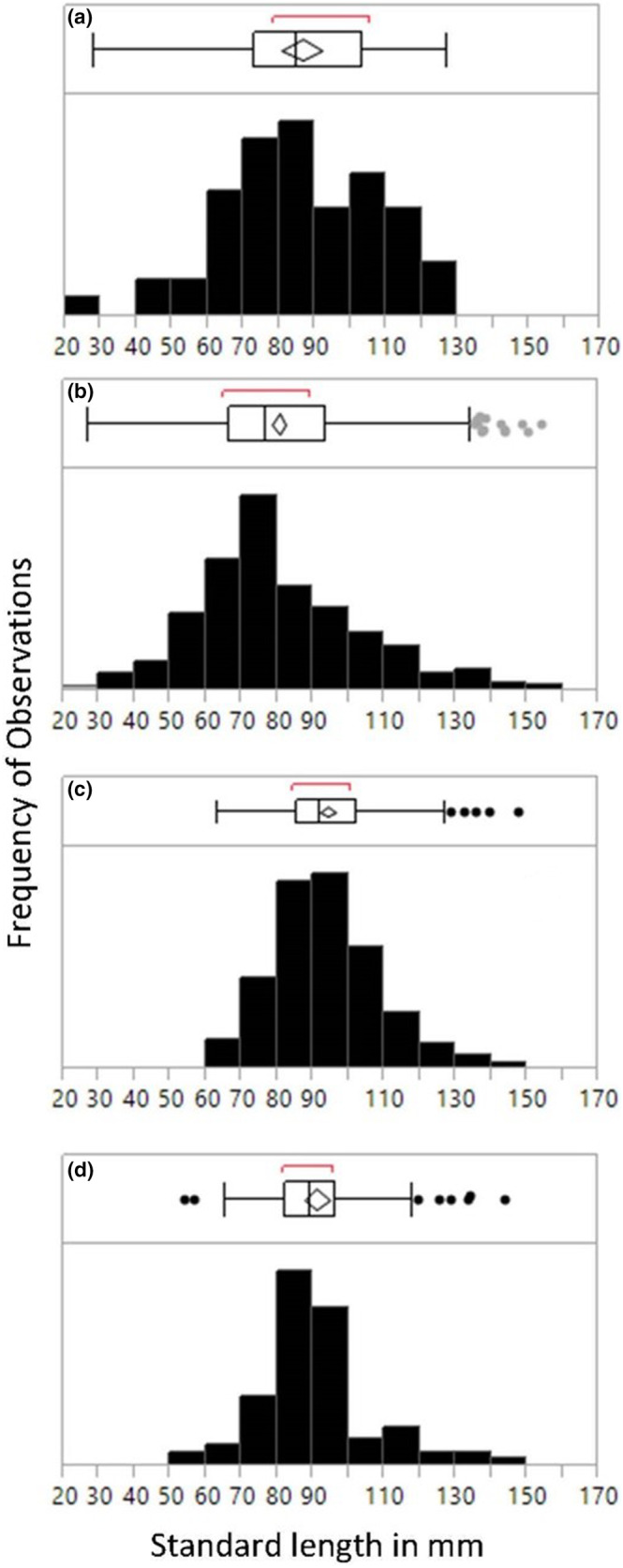
Size frequency distributions (standard length in mm) for all seahorses observed seasonally during the day (data from Masonjones et al., 2019) and seasonally during the night (present study). (a) Dry season (March) daytime fish (*n* = 56), (b) dry season nighttime fish (*n* = 528), (c) wet season (August) daytime fish (*n* = 183), (d) wet season nighttime fish (*n* = 99).

Size frequency distributions for the various time of day and season combinations differed in range and shape, with mean body length smaller in the dry season than during the wet season (Figure 6). The biggest difference in distribution shape was between night size distributions across seasons (Two‐Sample Kolmogorov–Smirnov test, Dstat = 0.222, Dcrit_0.01_ = 0.186, *p* < 0.01). Mean standard length differed by the time of day sampled and season (GLM, *X* ^2^ = 70.336, df = 3, *p* < .0001; Figure 6), with nighttime mean body size 10 mm smaller than daytime means. Larger seahorses were also documented during the dry season (*X* ^2^ = 21.651, df = 1, *p* < .0001) and during the daytime surveys (*X* ^2^ = 5.627, df = 1, *p* = .018), with no interaction effects (*X* ^2^ = 0.677, df = 1, *p* = .410). When restricting the analyses to adults above 65 mm (size at maturity for this population; Masonjones et al., 2019; GLM, *X* ^2^ = 27.956, df = 7, *p* = .0002; Table 1), differences were driven by the interaction between the time of day (*X* ^2^ = 5.051, df = 1, *p* = .025) and season (*X* ^2^ = 4.440, df = 1, *p* = .035), with no difference observed between males and females (*X* ^2^ = 0.01, df = 1, *p* = .99).

### 3.4 Habitat use and preference

#### 3.4.1 Seahorses

We investigated seahorse body posture, perch height, and holdfast use during night and day transects. Animals of both sexes were more likely to be upright during the night and prone during the day. Body posture, ranging from 0 (lying prone relative to the substrate) to 1 (fully upright relative to the substrate) differed significantly by time of day (GLM, *X* ^2^ = 131.90, df = 5, *p* < .0001; *X* ^2^ = 21.887, df = 1, *p* < .0001) but not by distance from shore (GLM, *X* ^2^ = 2.797, df = 2, *p* = .247). This pattern did not vary by sex (Wilcoxon test, *X* ^2^ = 2.181, df = 2, *p* = .336).

When compared between day and night transects, mean perch height was significantly higher during the night than during the day with fish located 286% vertically higher on holdfasts than during the night. When we investigated perch height relative to the time of day (GLM, *X* ^2^ = 580.015, df = 34, *p* < .0001), fish were significantly higher in the vegetation at night (*X* ^2^ = 166.942, df = 30, *p* < .0001), but there was no difference in perch height by the fish's sex or distance from shore (both *p* < .05).

**FIGURE 7:**
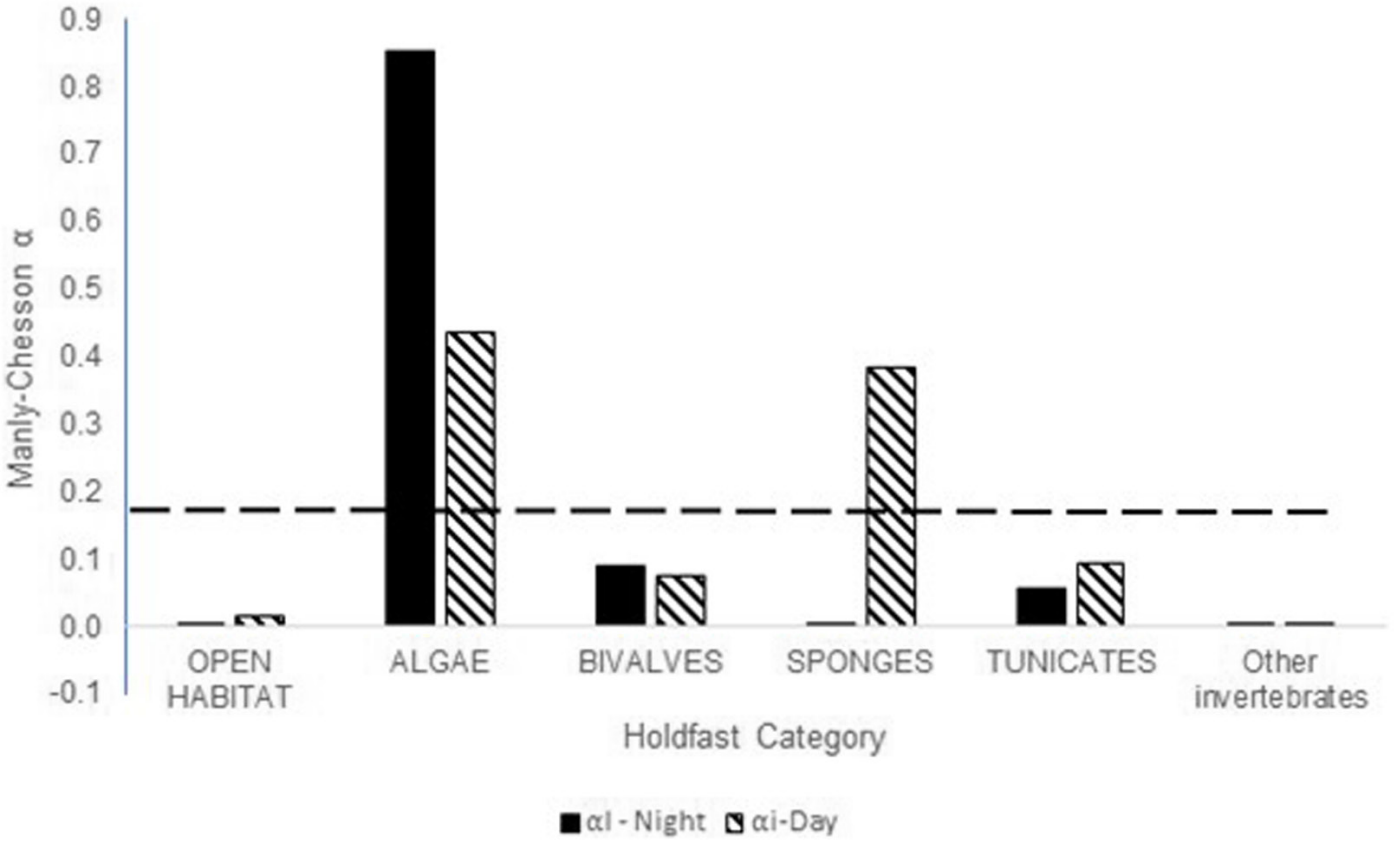
Manly–Chesson *⍺* scores identifying preference versus avoidance scores for holdfast types for seahorses. Twelve major benthic categories were identified, but seahorses used only the first five habitats as holdfasts. The dashed line indicates the expected frequency given the number of categories used in the analysis (*m* = 6) with values below the line indicating avoidance behavior and values above the line indicating the preference of habitat component.

Holdfast preference differed significantly between the combined effects of sex (M, F, J) and time of day (Nominal Logistic Fit, *X* ^2^ = 72.226, df = 39, *p* = .0062), but only time of day was significant and sex only has an influence if coded as part of an interaction with the time of day (*X* ^2^ = 42.481, df = 13, *p* < .0001; Table 2). For the three replicates that we repeated across the season, seasonal effects on holdfast use were not detected (Nominal Logistic Fit, global *X* ^2^ = 21.37, df = 8, *p* = .0062, Time of Day *X* ^2^ = 18.108, df = 4, *p* = .0012, Season *X* ^2^ = 2.551, df = 4, *p* = .6356). There were some holdfasts that seahorses appeared to avoid, independent of the time of day (Figure 7, displayed as Manly–Chesson ⍺ values at or close to zero), and open habitat was avoided when time was considered. Algae was strongly preferred both daytime and nighttime, whereas sponges were solely preferred during the day but were avoided at night.

**TABLE 2:** Table of benthic cover species by percent with holdfasts used by seahorses identified by count and frequency

Category	Percent benthic cover type in habitat	Species	Common name	Day – Number of seahorses on holdfast	Night – Number of seahorses on holdfast	Day %	Night %
Algae/Flowering plant	42.1%	*Laurencia* spp	Red algae	183	593	0.906	0.943
		*Hypnea musciformis*	Red algae	2	1	0.010	0.002
		*Caulerpa sertularioides*	Green feather algae	2	23	0.010	0.037
		Coralline Algae		0	2	0.000	0.003
		*Rhizophora mangle*	Red mangrove	0	2	0.000	0.003
Bivalves	2.6%	*Laevicardium pictum*	Ravenel's egg cockle	1	0	0.005	0.000
		*Chione elevata*	Cross‐barred venus clam	1	1	0.005	0.002
		*Pinctata radiata*	Atlantic pearl oyster	0	3	0.000	0.005
Other invertebrates	6.1%	*Iricina strobalina*	Black ball sponge	5	0	0.025	0.000
		*Ecteinascidia turbinata*	Mangrove tunicate	1	1	0.005	0.002
Open habitat	29.4%	Flocculant		6	1	0.030	0.002
	19.4%	Sand		1	2	0.005	0.003

*Note*: Remaining 0.4% of benthic cover total is made of cyanobacteria and seahorses themselves.

#### 3.4.2 Potential predators

There was no linear relationship between the density of shoreline predators and total seahorse density (*F*_1,13_ = 0.0009, *p* = .976), even when restricted to the nearshore transect region closest to the shoreline (*F*_1,13_ = 0.953, *p* = .347; Table 3). There was also no relationship between shoreline predator density and the transect predator density (both nocturnal and diurnal; nocturnal: *F*_1,13_ = 0.727, *p* = .409, diurnal: *F*_1,13_ = 0.324, *p* = .579).

**TABLE 3:** Tallies of predators surveyed across the study, both in the 15 transects (Total area surveyed 3600 m^2^) and along the shoreline (linear distance surveyed 381 m, with average shoreline depth 2 m)

Potential predators	Transects		Shoreline
	Day	Night	Day
*Maguimithrax spinosissimus*	5	53	92
*Octopus briareus*	5	8	5
*Epinephelus striatu*s and other large fish predators	0	0	7
Predator density (m^2^ or m)	0.003 (0.0021)	0.027 (0.0071)	0.358 (0.10)

Predator densities on transects were significantly higher during the night (Wilcoxon Signed Rank test, *S* = 55.00, *n* = 15, *p* = .0007; Table 3). No significant difference in predator density was observed between seasons (Repeated measures MANOVA, *F*_1,4_ = 1.5, *p* = .288) although more animals were surveyed at night in the wet season compared with dry. Predators on transects did not differ in their densities relative to the distance from shore (Repeated Measures MANOVA, *F*_1,28_ = 0.336, *p* = .566). Encountered octopuses were either moving through the landscape or in dens (cavities or depressions with evidence of shell debris around them) and we did not observe any actively eating or handling prey during the day or night. Seventy‐one percent of crabs observed were foraging on algae and the rest were not foraging; we observed no other food types during feeding.

## 4 DISCUSSION

This is one of the first studies to systematically document diel changes in the population density, juvenile recruitment, behavior, and holdfast preference in a wild seahorse. Sweetings Pond *H. erectus* displayed dramatically increased densities during the night, far higher than densities observed in previous daytime studies of this system and higher than that seen in other seahorse populations worldwide (Foster & Vincent, 2004; Masonjones et al., 2019; Masonjones & Rose, 2019). In addition to the higher densities, we also observed behaviors that increased the visibility of seahorses, including a nocturnal shift of animals moving shallower in the water column paired with upright body postures. This change in behavior increased the detectability for all members of the population, revealing a robust population of juveniles during the dry season, which indicates that the recruitment of the population has yet to be measured by the daytime surveys. Our findings are important for the broader understanding of seahorse ecology, which we address below for each major result. Additionally, our system highlights potential sampling bias created by only surveying populations during the day, which has critical implications across a diverse set of species and ecosystems. The conclusions from this study indicate the need for sampling populations at times that best reflect the biology of the species studied to assess and manage them for conservation targets.

### 4.1 Population density

The drastic differences in diel densities have key ecological and management implications for seahorse populations. Daytime densities in this study mirror the densities we previously reported in the northern section of the lake, obtained through exhaustive sampling by actively combing through the vegetation for seahorses (Masonjones et al., 2019). As a result, the observation that we obtained similar numbers with a thorough visual inspection of the habitat, without any direct contact with the fish or combing through the macroalgae looking for them, demonstrates consistency in the findings between the two studies. While the daytime densities recorded in Sweetings Pond are higher than the worldwide average seahorse density (Masonjones & Rose, 2019), the nighttime density from this study is one of the highest ever reported for seahorses, especially from randomly established transects that are not on artificial habitats (Correia et al., 2013; Simpson et al., 2019). The drastic difference between the day and night densities did not change between the dry and wet seasons suggesting this phenomenon is not solely due to seasonal recruitment.

This study is one of the few to document nocturnality in seahorses and therefore, the dramatic increase in nighttime densities indicates that we are potentially miscalculating population estimates by only sampling during the day. Because the extent of this bias is currently unknown in seahorses, we must base management decisions on daytime estimates across populations. Given the unique nature of Sweetings Pond being an isolated population under relaxed predatory selection, these nocturnal behaviors could be rare for seahorses or they may be more widespread. Broader nocturnal surveys across other *H. erectus* populations and other seahorse species are needed to assess whether this trend is common across all members of the *Hippocampus* genus.

### 4.2 Reproduction

#### 4.2.1 Sex ratios

The ratio of adult males to adult females (the adult sex ratio, ASR) is often used as an assessment of the mating system of a species. Seahorse species studied to date display genetically monogamous mating systems (Jones et al., 1998; Jones & Avise, 2001; Rose et al., 2014) with predominantly even to female‐biased sex ratios (0.457, daytime global seahorse sex ratio; Masonjones & Rose, 2019). In this Sweetings Pond lined seahorse population, both sex ratio and density shift between daytime and nighttime but not seasonally, suggesting different sexually selective landscapes in the shallows across each 24‐h cycle. The daytime sex ratio of 0.80 indicates a strongly male‐biased population that is similar to the results observed in previous work in Sweetings Pond, where roughly two males were observed for each female (Masonjones et al., 2019). The nocturnal population in Sweetings Pond exhibits a more even sex ratio of 0.61 that is approaching that of a typical monogamous species but is still significantly male‐biased compared with sex ratios observed in other seahorse species.

Given the robust size of this seahorse population and the consistent bias towards males regardless of the season across the previous 6 years of sampling, the Sweetings Pond *H. erectus* presents the opportunity to investigate mating system variation. Although there was no detectable effect of distance to shore on adult seahorse density, both sexes tended to congregate in deeper areas on a select few transects (Figure S1a,b). Varying both sex ratio and population density in this way can impact the reproductive and foraging behaviors of animals (Aronsen et al., 2013; Ewen et al., 2011; Kokko & Rankin, 2006; Vahl et al., 2013). For example, higher densities of males in species with male–male competition can lead to increases in the vigor and duration of contests but also to lower levels of competition in some contexts (de Jong et al., 2009; Griskevicius et al., 2012; Masonjones & Rose, 2019; Naud et al., 2009). In seahorses, the additional males can suppress reproduction due to escalated male–male competition affecting the success of egg transfers from females (Masonjones & Rose, 2019). The consistent male bias in Sweetings Pond shows the opportunity for a shift in the mating system in this population. For example, in species with traditional sex roles, higher numbers of males relative to females can increase the selection of males and rapidly accelerate the development of sexually selected traits, including nuptial coloration, larger body sizes, and other ornaments (Forsgren et al., 2004; Puechmaille et al., 2014; Wacker et al., 2013).

#### 4.2.2 Females

We observed far more females at night, which suggests that females are either more cryptic than males during the day or are migrating from the depths to the shallows at night. Because a more even sex ratio with more females was observed during exhaustive searches of this region in the dry season, there is support for the hypothesis that females spend their day burrowing in the algae, resulting in more cryptic behaviors than males. However, an equally plausible explanation for the lack of females during daytime sampling is that they move to deeper habitats during the day. Although a small sample size, the two deeper transects not included in our analysis had only large females (*n* = 6, mean body size 95.83 (8.88) mm), so there is the potential that females have different daytime habitat preferences for deeper water or are more mobile than males during the day (Figure S1a,b). Evidence from other studies suggests that seahorse females often possess larger home ranges and thus move more than males, which has been seen in the tiger‐tail seahorse, *H. comes* (Perante, 2002), White's seahorse, *H. whitei* (Foster & Vincent, 2004; Harasti et al., 2014a, 2014b; Vincent et al., 2005; Vincent & Sadler, 1995), short‐snouted seahorse, *H. breviceps* (Moreau & Vincent, 2004), and other species (Foster & Vincent, 2004; Harasti et al., 2014a, 2014b; Vincent et al., 2005; Vincent & Sadler, 1995). In addition, *H. reidi* females were found to be the most active group whereas gravid males were the least active (Freret‐Meurer et al., 2012), mirroring our observation that females and nongravid males were not observed as often during the day, whereas gravid males were the most commonly seen group.

#### 4.2.3 Body size shifts reflect juvenile recruitment

Across multiple lines of evidence, one of our most important discoveries was the abundance of newborns and juveniles at night during the dry season. This was confirmed by our 11% decrease in average body size in nighttime samples when localized to the shallowest parts of transects close to shore (Figure S1c). Although we have noted in previous research in Sweetings Pond and confirmed here that gravid males are more common during the dry season (Masonjones et al., 2019), we rarely detect juveniles below 40 mm during daytime sampling. This shift indicates that juveniles are emerging from the vegetation at night and that the dominant period of juvenile recruitment is in the dry season, with less reproduction occurring in the wet season. Additionally, the observation of a size shift in the population between the time of day and season persisted despite the removal of juveniles (defined as fish smaller than 65 mm) from the analysis. This indicates that younger adults, other than newborns are also present more often at night than during the day and that the population is composed of generally smaller animals during the dry season.

The greater representation of pregnant males and juveniles in Sweetings Pond *H. erectus* both occurring during the dry months is also seen in *H. guttulatus* when both juveniles and pregnant males peaked during the same month of June (Gristina et al., 2017). Little information exists on juvenile recruitment patterns in seahorses and juveniles are rarely observed before their settlement as mid‐sized juveniles into the habitats where adults are observed (Curtis et al., 2017; Foster & Vincent, 2004). It is also possible that juveniles in other systems are rarely observed because they are often dispersed by water currents from adult areas via rafting, as seen in a range of seahorse species (Bertola et al., 2020; Boehm et al., 2013; Luzzatto et al., 2013; Teske et al., 2007). Because it is an enclosed body of water with minimal hydrodynamical movement, Sweetings Pond seahorse juveniles may not engage in rafting in this system, but this has not yet been assessed.

### 4.3 Behavioral changes in habitat use

At night, seahorses displayed higher perch heights and upright body postures and changes in holdfast preferences. During the day, Sweetings Pond seahorses displayed more cryptic behaviors, with animals lying prone on the substrate, similar to the thanatosis (death‐like horizontal posture) behaviors observed in the slender seahorse (*Hippocampus reidi*, Freret‐Meurer et al., 2017). Factors driving this shift in the water column and changing body position remain unclear. In the only other study of seahorses at night (*H. comes* on reefs in the Philippines), animals were observed up at the top of the reef crest at night and were not visible during the day across the 24‐month study (*n* = 32 seahorses; Perante, 2002). In that study, nocturnality was hypothesized to be driven by human fishing practices, but it is possible based on the present study that this nocturnal vertical migration might be more common in seahorses than previously thought. However, crypsis during the day could suggest a predator avoidance behavior, and so, understanding the potential predator pool of the system is important. It is also possible that seahorses move up in the water column to better exploit ambient light and continue feeding, given observations of full guts both during the day and at night (Masonjones et al., unpublished). In European minnows, nighttime feeding is the primary foraging mode except in fish with malnutrition, so nocturnal foraging may be critical for fish (Metcalfe & Steele, 2001).

This work is the first to report holdfast preferences in this unique high seahorse‐density Bahamian habitat. Of the potential holdfasts in the pond's northern region, we found that macroalgae were both the most abundant and most strongly preferred holdfast independent of time of day, whereas sponges were the second most preferred holdfast during the day but were rarely used at night. Seahorses using the sponges during the day were typically darkened in coloration and exhibited thanatosis behaviors, which could be due to increased predation. In *H. whitei*, adults prefer sponges and soft coral as holdfasts during the day, some of the taller holdfasts in the areas where they are found, while juveniles prefer gorgonians (Harasti et al., 2014a, 2014b). In Sweetings Pond, juveniles were found in the shallows where the first 15 m of transects closest to shore had the greatest amount of macroalgae. The preference for macroalgae parallels those found in *H. comes* where juveniles were most abundant on macroalgae, while adults were found equally on macroalgae and corals (Morgan & Vincent, 2007). Gristina et al. (2017) documented that *H. guttulatus* juveniles were found on macroalgae in the shallows with adults deeper in the water column on preferred holdfasts of *Cladophora prolifera* and poles in an oyster farm. These examples indicate that some seahorse species have juveniles and adults that prefer separate microhabitats, whereas other species, like Sweetings Pond *H. erectus*, differ, with juveniles having a smaller but overlapping range with adults. Ongoing spatial analysis of this data set indicates that fish on transects were found to have dispersed distributions in the landscape during the day, indicating a distribution often associated with territoriality or distinct home ranges but were either randomly distributed or clustered on the nocturnal transects (Elson, 2019).

### 4.4 Potential predators

Due to increases in their population densities at night, the potential exists for Caribbean reef octopus (*Octopus briareus*) and West Indian spider crabs (*Maguimithrax spinosissimus*) to be nocturnal seahorse predators in Sweetings Pond, and thus impact seahorse behavior. In addition, large introduced Nassau grouper (*Epinephelus striatus*) are also found in the system and have been observed during the day. Given their gape size, the lack of many food species fish, and the abundance of seahorses, they are also candidate predators (Aronson, 1985). Octopus range in density across space and seasons (Aronson, 1985; O'Brien et al., 2020) and have been demonstrated in Australian systems to be active seahorse predators (Harasti et al., 2014a, 2014b). Seahorses and pipefishes are included in the diets of a wide range of species, including grouper, octopus, and crabs (Kleiber et al., 2011). Although the specific species found in the pond have never been described to actively hunt *H. erectus*, it is conceivable they could. Spider crabs (*Maguimithrax spinosissimus*) have been observed feeding on seahorses at the site (personal observation); however, no evidence of predatory behavior of crabs on seahorses was observed in this study, with crabs and seahorses regularly in close physical contact. These crabs feed predominantly at night, which corresponds to seahorse peak conspicuousness, where they move to the top of the benthic canopy.

A second potential explanation for the Sweetings Pond seahorses laying flat during the day could be in response to avian predators. Surveys of predators indicate that seahorse and pipefish populations found in higher abundances have greater opportunistic bird predation (Kleiber et al., 2011). The high‐density population in Sweetings Pond and the isolation of the population from oceanic habitats could potentially serve as an optimal feeding ground for avian predators, leading to intense selective pressures on the seahorses' perch height and the increased daytime crypsis in the pond. Given that seahorses are more accessible along the shoreline in shallow water, we would expect that if this were a factor the nearshore halves of the transects would exhibit fish lower in the macroalgal canopy during the day, with more prone body postures. However, there was no difference with distance to shore either at night or during the day for these two behavioral variables. There could be a selective advantage for the seahorses adapting thanatosis behaviors, regardless of the depth, because of the limited turbidity and pristine water clarity in the isolated pond.

### 4.5 Future directions for Sweetings Pond

This study identifies several new lines of inquiry to help build a complete picture of this unique ecosystem. Our findings reveal the potential misconceptions of the population dynamics of specific species that we make as biologists when only investigating questions at one time of day. Some of the hypotheses for the seahorse's increased detectability and upright posture at night include predator avoidance, feeding, and social behaviors such as courtship. The detection of the increase in potential predators and seahorses both observed at night indicate that more work is needed to survey nighttime community interactions in the system. Previous studies in other fish species indicate that nocturnal behaviors can result from lower food availability. However, the opposite can also be true, where well‐fed fish are active at night, and the poorly sustained fishes feed throughout the day (Metcalfe & Steele, 2001). Future studies focused on food availability, and the seahorses' feeding will address this question in our system.

Lastly, the potential connection between seahorses' reproductive biology/mating behavior and their increased nocturnal detectability and larger congregations remains unclear at night. Courtship displays have yet to be fully documented in the Sweetings Pond population, even though there has been extensive surveying at dawn, dusk, daytime, and nighttime under several stages of the lunar cycle, with brief observations of courtship behavior observed from 4 am through late afternoon. Future work will focus on documenting mating displays during the height of their seasonal breeding that was discovered during this study to increase the probability of these events occurring and being recorded. Beyond courtship behaviors, molecular approaches are needed to understand the mating system of this subpopulation. Although it is highly unlikely that males receive eggs from more than one female in a breeding event based on the morphological restrictions posed by the seahorse ovary (Sogabe et al., 2008), given the large number of males in the population, it is possible that fish may switch mates between breeding events. However, recent computational approaches suggest, that there is an interplay between female dispersion in the landscape (as we observed during the day) and male‐biased populations that support the evolution and maintenance of monogamy (Gomes et al., 2018). As a result, Sweetings Pond is an excellent model in which to investigate the relationship between a population's social system and the potential for sexual selection to shape reproductive behaviors and mating system. This is a particularly important question given the Vulnerable Red List status of this species and the implications that reproduction has on population estimates and conservation management strategies (Pollom, 2017).

## 5 CONCLUSIONS

The implications from our findings and the lessons learned from our nocturnal surveys provided valuable insight into the need for a more critical assessment of survey methods used in other systems. Reflecting on our study, the results indicated when and how you census your population matters a lot. An exploratory night dive made it abundantly clear why this population, with an unusual sex ratio, required more investigation and led to the discovery of these unique nocturnal behaviors and extreme diel density fluctuations. Nocturnal “blindness” could be one of the largest overlooked components of sampling designs and it is supported by that the fact that we know very little about the nocturnal biology for the majority of species, particularly those requiring conservation measures. In some cases, this discrepancy is because the species itself is understudied, but in many cases, it is often due to our diurnal bias or because the ease of working during the day or sampling restrictions drove the science and limited the experimental design.

Evidence from across systems supports the assertion that population surveys need to include nighttime sampling. Studies of other fish species using acoustic techniques and trawling to assess fish populations have had mixed results sampling during the day compared with at night, with some species or water bodies with higher densities during the day and others higher at night (Draštík et al., 2009; Yule et al., 2007). Additionally, some studies indicate the importance of surveying fish populations at night for accurate counts, often because of differences in fish habitat use between day and night and the challenges of surveying fish in more complex habitats like coral reefs (Fox & Bellwood, 2011). An advancement in technology will also likely provide more opportunities to survey nocturnal populations as low‐light cameras and underwater drones. For example, the recent advent of techniques like BRUV's (baited remote underwater videos) underwater and camera traps for terrestrial communities that can capture images in low‐light settings at night have helped to illustrate just how important nocturnal dynamics are within species and across habitats (Harvey et al., 2012, 2021; Swann & Perkins, 2014). Further magnifying the need to survey at night, global increases in daytime human activities are shifting some animal populations to a more nocturnal lifestyle (Gaynor et al., 2018). This shift has led to key ecosystem effects like prey changes to nocturnal species, alterations in foraging patterns, and changing competition regimes, all factors that dramatically affect both the functioning of these ecosystems and the management of populations within them.

## AUTHOR CONTRIBUTIONS

**Heather D. Mason:** Conceptualization (equal); data curation (lead); formal analysis (lead); funding acquisition (equal); investigation (equal); methodology (equal); project administration (equal); software (lead); writing – original draft (lead); writing – review and editing (equal). **Emily Rose:** Conceptualization (equal); funding acquisition (equal); investigation (supporting); methodology (equal); writing – review and editing (equal). **Jessica Elson Gonzalez:** Formal analysis (supporting); funding acquisition (equal); investigation (supporting); methodology (equal); writing – review and editing (equal). **Duncan A. O'Brien:** Investigation (supporting); writing – review and editing (supporting).

## CONFLICT OF INTEREST

The authors declare that there are no competing interests.

## Supporting information


Appendix S1.

Figure S1.

## Data Availability

The data will be archived and made available on Dryad upon acceptance of the manuscript at the following location: DOI https://doi.org/10.5061/dryad.z612jm6g7. The temporary link to the data set is at https://datadryad.org/stash/share/0EJDaWjl_182k7xc_SHEEjlCzXfHqKtS2A7nKaz4BtA.
