# Supplementary material for: Nocturnal surveys of lined seahorses reveal increased densities and seasonal recruitment patterns
Source: Ecol Evol. 2023 Jan 11;13(1):e9573. doi: 10.1002/ece3.9573 (PMC9834011; doi:10.1002/ece3.9573)
Supplement: Supplementary file 1 — Appendix S1. [file ECE3-13-e9573-s001.pdf]

Appendices I, II and III

valid from 22 June 2022

Interpretation

1. Species included in these Appendices are referred to:

   a) by the name of the species; or
   b) as being all of the species included in a higher taxon or designated part thereof.

2. The abbreviation “spp.” is used to denote all species of a higher taxon.

3. Other references to taxa higher than species are for the purposes of information or classification only. The common names included after the scientific names of families are for reference only. They are intended to indicate the species within the family concerned that are included in the Appendices. In most cases this is not all of the species within the family.

4. The following abbreviations are used for plant taxa below the level of species:

   a) “ssp.” is used to denote subspecies; and
   b) “var(s).” is used to denote variety (varieties).

5. As none of the species or higher taxa of FLORA included in Appendix I is annotated to the effect that its hybrids shall be treated in accordance with the provisions of Article III of the Convention, this means that artificially propagated hybrids produced from one or more of these species or taxa may be traded with a certificate of artificial propagation, and that seeds and pollen (including pollinia), cut flowers, seedling or tissue cultures obtained *in vitro*, in solid or liquid media, transported in sterile containers of these hybrids are not subject to the provisions of the Convention.

6. The names of the countries in parentheses placed against the names of species in Appendix III are those of the Parties submitting these species for inclusion in this Appendix.

7. When a species is included in one of the Appendices, the whole, live or dead, animal or plant is included. In addition, for animal species listed in Appendix III and plant species listed in Appendix II or III, all parts and derivatives of the species are also included in the same Appendix unless the species is annotated to indicate that only specific parts and derivatives are included. The symbol # followed by a number placed against the name of a species or higher taxon included in Appendix II or III refers to a footnote that indicates the parts or derivatives of animals or plants that are designated as 'specimens' subject to the provisions of the Convention in accordance with Article I, paragraph (b), subparagraph (ii) or (iii).

8. The terms and expressions below, used in annotations in these Appendices, are defined as follows:

   Extract

   Any substance obtained directly from plant material by physical or chemical means regardless of the manufacturing process. An extract may be solid (e.g. crystals, resin, fine or coarse particles), semi-solid (e.g. gums, waxes) or liquid (e.g. solutions, tinctures, oil and essential oils).

   Finished musical instruments

   A musical instrument (as referenced by the Harmonized System of the World Customs Organization, Chapter 92; musical instruments, parts and accessories of such articles) that is ready to play or needs only the installation of parts to make it playable. This term includes antique instruments (as defined by the Harmonized System codes 97.05 and 97.06; Works of art, collectors' pieces and antiques).

Finished musical instrument accessories

A musical instrument accessory (as referenced by the Harmonized System of the World Customs Organization, Chapter 92; musical instruments, parts and accessories of such articles) that is separate from the musical instrument, and is specifically designed or shaped to be used explicitly in association with an instrument, and that requires no further modification to be used.

Finished musical instrument parts

A part (as referenced by the Harmonized System of the World Customs Organization, Chapter 92; musical instruments, parts and accessories of such articles) of a musical instrument that is ready to install and is specifically designed and shaped to be used explicitly in association with the instrument to make it playable.

Finished products packaged and ready for retail trade

Products, shipped singly or in bulk, requiring no further processing, packaged, labelled for final use or the retail trade in a state fit for being sold to or used by the general public.

Powder

A dry, solid substance in the form of fine or coarse particles.

Shipment

Cargo transported under the terms of a single bill of lading or air waybill, irrespective of the quantity or number of containers or packages; or pieces worn, carried or included in personal baggage.

Ten (10) kg per shipment

For the term "10 kg per shipment", the 10 kg limit should be interpreted as referring to the weight of the individual portions of each item in the shipment made of wood of the species concerned. In other words, the 10 kg limit is to be assessed against the weight of the individual portions of wood of Dalbergia/Guibourtia *species contained in each item of the shipment, rather than against the total weight of the shipment.*

Transformed wood

Defined by Harmonized System code 44.09: Wood (including strips, friezes for parquet flooring, not assembled), continuously shaped (tongued, grooved, v-jointed, beaded or the like) along any edges, ends or faces, whether or not planed, sanded or end-jointed.

Woodchips

Wood that has been reduced to small pieces.

	Appendices		
	I	II	III
**FAUNA (ANIMALS)**<br>**PHYLUM CHORDATA**<br>**CLASS MAMMALIA (MAMMALS)**			
ARTIODACTYLA			
Antilocapridae Pronghorns			
	Antilocapra americana (Only the population of Mexico; no other population is included in the Appendices)		
Bovidae Antelopes, cattle, duikers, gazelles, goats, sheep, etc.			
	Addax nasomaculatus		
		Ammotragus lervia	
			Antilope cervicapra (Nepal, Pakistan)
	Bos gaurus (Excludes the domesticated form, which is referenced as *Bos frontalis*, and is not subject to the provisions of the Convention)		
	Bos mutus (Excludes the domesticated form, which is referenced as *Bos grunniens*, and is not subject to the provisions of the Convention)		
	Bos sauveli		
			Boselaphus tragocamelus (Pakistan)
			Bubalus arnee (Excludes the domesticated form, which is referenced as *Bubalus bubalis* and is not subject to the provisions of the Convention) (Nepal)
	Bubalus depressicornis		
	Bubalus mindorensis		
	Bubalus quarlesi		
		Budorcas taxicolor	
		Capra caucasica	
	Capra falconeri		

	Appendices<br>I	 II	 III
			Capra hircus aegagrus (Specimens of the domesticated form are not subject to the provisions of the Convention) (Pakistan)
			Capra sibirica (Pakistan)
	Capricornis milneedwardsii		
	Capricornis rubidus		
	Capricornis sumatraensis		
	Capricornis thar		
		Cephalophus brookei	
		Cephalophus dorsalis	
	Cephalophus jentinki		
		Cephalophus ogilbyi	
		Cephalophus silvicultor	
		Cephalophus zebra	
		Damaliscus pygargus pygargus	
			Gazella bennettii (Pakistan)
	Gazella cuvieri		
			Gazella dorcas (Algeria, Tunisia)
	Gazella leptoceros		
	Hippotragus niger variani		
		Kobus leche	
	Naemorhedus baileyi		
	Naemorhedus caudatus		
	Naemorhedus goral		
	Naemorhedus griseus		
	Nanger dama		
	Oryx dammah		
	Oryx leucoryx		
		Ovis ammon	
		Ovis arabica	
		Ovis bochariensis	
		Ovis canadensis (Only the population of Mexico; no other population is included in the Appendices)	

	Appendices I	II	III
		Ovis collium	
		Ovis cycloceros	
		Ovis darwini	
	Ovis gmelini (Only the population of Cyprus; no other population is included in the Appendices)		
	Ovis hodgsoni		
		Ovis jubata	
		Ovis karelini	
	Ovis nigrimontana		
		Ovis polii	
		Ovis punjabiensis	
		Ovis severtzovi	
	Ovis vignei		
	Pantholops hodgsonii		
		Philantomba monticola	
			Pseudois nayaur (Pakistan)
	Pseudoryx nghetinhensis		
		Rupicapra pyrenaica ornata	
		Saiga borealis (A zero export quota for wild specimens traded for commercial purposes)	
		Saiga tatarica (A zero export quota for wild specimens traded for commercial purposes)	
			Tetracerus quadricornis (Nepal)

	Appendices I	II	III
Camelidae Camels, guanacos, vicunas			
	Vicugna vicugna [Except the populations of: Argentina (the populations of the Provinces of Jujuy, Catamarca and Salta, and the semi-captive populations of the Provinces of Jujuy, Salta, Catamarca, La Rioja and San Juan), Chile (populations of the region of Tarapacá and of the region of Arica and Parinacota), Ecuador (the whole population), Peru (the whole population) and the Plurinational State of Bolivia (the whole population), which are included in Appendix **II**]	***Lama guanicoe***<br><br>***Vicugna vicugna*** [Only the populations of Argentina (the populations of the Provinces of Jujuy, Catamarca and Salta, and the semi-captive populations of the Provinces of Jujuy, Salta, Catamarca, La Rioja and San Juan), Chile (populations of the region of Tarapacá and of the region of Arica and Parinacota), Ecuador (the whole population), Peru (the whole population) and the Plurinational State of Bolivia (the whole population); all other populations are included in Appendix **I**] [1]	

[1] For the exclusive purpose of allowing international trade in fibre from vicuñas (*Vicugna vicugna*) and their derivative products, only if the fibre comes from the shearing of live vicuñas. Trade in products derived from the fibre may only take place in accordance with the following provisions:

a) Any person or entity processing vicuña fibre to manufacture cloth and garments must request authorization from the relevant authorities of the country of origin (Countries of origin: The countries where the species occurs, that is, Argentina, Bolivia, Chile, Ecuador and Peru) to use the "vicuña country of origin" wording, mark or logo adopted by the range States of the species that are signatories to the Convention for the Conservation and Management of the Vicuña.

b) Marketed cloth or garments must be marked or identified in accordance with the following provisions:

   i) For international trade in cloth made from live-sheared vicuña fibre, whether the cloth was produced within or outside of the range States of the species, the wording, mark or logo must be used so that the country of origin can be identified. The VICUÑA [COUNTRY OF ORIGIN] wording, mark or logo has the format as detailed below:

	Appendices I	II	III
Cervidae Deer, huemuls, muntjacs, pudus			
	Axis calamianensis		
	Axis kuhlii		
			Axis porcinus (Except the subspecies included in Appendix I) (Pakistan)
	Axis porcinus annamiticus		
	Blastocerus dichotomus		
		Cervus elaphus bactrianus	
			Cervus elaphus barbarus (Algeria, Tunisia)
	Cervus elaphus hanglu		
	Dama dama mesopotamica		
	***Hippocamelus* spp.**		
			Mazama temama cerasina (Guatemala)
	Muntiacus crinifrons		
	Muntiacus vuquangensis		
			Odocoileus virginianus mayensis (Guatemala)
	Ozotoceros bezoarticus		

VICUÑA [PAÍS DE ORIGEN]

This wording, mark or logo must appear on the reverse side of the cloth. In addition, the selvages of the cloth must bear the words VICUÑA [COUNTRY OF ORIGIN].

ii) For international trade in garments made from live-sheared vicuña fibre, whether the garments were produced within or outside of the range States of the species, the wording, mark or logo indicated in paragraph b) i) must be used. This wording, mark or logo must appear on a label on the garment itself. If the garments are produced outside of the country of origin, the name of the country where the garment was produced should also be indicated, in addition to the wording, mark or logo referred to in paragraph b) i).

c) For international trade in handicraft products made from live-sheared vicuña fibre produced within the range States of the species, the VICUÑA [COUNTRY OF ORIGIN] - ARTESANÍA wording, mark or logo must be used as detailed below:

VICUÑA [PAÍS DE ORIGEN] - ARTESANÍA

d) If live-sheared vicuña fibre from various countries of origin is used for the production cloth and garments, the wording, mark or logo of each of the countries of origin of the fibre must be indicated, as detailed in paragraphs b) i) and ii).

e) All other specimens shall be deemed to be specimens of species listed in Appendix I and the trade in them shall be regulated accordingly.

	Appendices<br>I	 II	 III
	Pudu puda<br>***Rucervus duvaucelii***<br>***Rucervus eldii***	***Pudu mephistophiles***	
Giraffidae Giraffes			
		Giraffa camelopardalis	
Hippopotamidae Hippopotamuses			
		Hexaprotodon liberiensis<br>***Hippopotamus amphibius***	
Moschidae Musk deer			
	***Moschus* spp.** (Only the populations of Afghanistan, Bhutan, India, Myanmar, Nepal and Pakistan; all other populations are included in Appendix II)	***Moschus* spp.** (Except the populations of Afghanistan, Bhutan, India, Myanmar, Nepal and Pakistan, which are included in Appendix I)	
Suidae Babirusa, hogs, pigs			
	Babyrousa babyrussa<br>***Babyrousa bolabatuensis***<br>***Babyrousa celebensis***<br>***Babyrousa togeanensis***<br>***Sus salvanius***		
Tayassuidae Peccaries			
	Catagonus wagneri	**Tayassuidae spp.** (Except the species included in Appendix I and the populations of *Pecari tajacu* of Mexico and the United States of America, which are not included in the Appendices)	
CARNIVORA			
Ailuridae Red pandas			
	Ailurus fulgens		
Canidae Dogs, foxes, wolves			
			Canis aureus (India)

	Appendices I	II	III
	Canis lupus (Only the populations of Bhutan, India, Nepal and Pakistan; all other populations are included in Appendix II. Excludes the domesticated form and the dingo which are referenced as *Canis lupus familiaris* and *Canis lupus dingo*, respectively, which are not subject to the provisions of the Convention)		
		Canis lupus (Except the populations of Bhutan, India, Nepal and Pakistan, which are included in Appendix I. Excludes the domesticated form and the dingo which are referenced as *Canis lupus familiaris* and *Canis lupus dingo*, respectively, which are not subject to the provisions of the Convention)	
		Cerdocyon thous	
		Chrysocyon brachyurus	
		Cuon alpinus	
		Lycalopex culpaeus	
		Lycalopex fulvipes	
		Lycalopex griseus	
		Lycalopex gymnocercus	
	Speothos venaticus		
			Vulpes bengalensis (India)
		Vulpes cana	
			Vulpes vulpes griffithi (India)
			Vulpes vulpes montana (India)
			Vulpes vulpes pusilla (India)
		Vulpes zerda	
Eupleridae Fossa, falanouc, Malagasy civets			
		Cryptoprocta ferox	
		Eupleres goudotii	
		Fossa fossana	

	Appendices I	II	III
Felidae Cats			
		**Felidae spp.** [Except the species included in Appendix I. Excludes specimens of the domesticated form, which are not subject to the provisions of the Convention. For ***Panthera leo*** (African populations): a zero annual export quota is established for specimens of bones, bone pieces, bone products, claws, skeletons, skulls and teeth removed from the wild and traded for commercial purposes. Annual export quotas for trade in bones, bone pieces, bone products, claws, skeletons, skulls and teeth for commercial purposes, derived from captive breeding operations in South Africa, will be established and communicated annually to the CITES Secretariat.]	
	Acinonyx jubatus (Annual export quotas for live specimens and hunting trophies are granted as follows: Botswana: 5; Namibia: 150; Zimbabwe: 50. The trade in such specimens is subject to the provisions of Article III of the Convention)		
	Caracal caracal (Only the population of Asia; all other populations are included in Appendix II)		
	Catopuma temminckii		
	Felis nigripes		
	Herpailurus yagouaroundi (Only the populations of Central and North America; all other populations are included in Appendix II)		
	Leopardus geoffroyi		
	Leopardus guttulus		
	Leopardus jacobita		
	Leopardus pardalis		

	Appendices<br>I	II	III
	Leopardus tigrinus<br>***Leopardus wiedii***<br>***Lynx pardinus***<br>***Neofelis diardi***<br>***Neofelis nebulosa***<br>***Panthera leo*** (Only the populations of India; all other populations are included in Appendix II)<br>***Panthera onca***<br>***Panthera pardus***<br>***Panthera tigris***<br>***Panthera uncia***<br>***Pardofelis marmorata***<br>***Prionailurus bengalensis bengalensis*** (Only the populations of Bangladesh, India and Thailand; all other populations are included in Appendix II)<br>***Prionailurus planiceps***<br>***Prionailurus rubiginosus*** (Only the population of India; all other populations are included in Appendix II)<br>***Puma concolor*** (Only the populations of Costa Rica and Panama; all other populations are included in Appendix II)		

	Appendices I	II	III
Herpestidae Mongooses			
			Herpestes edwardsi (India, Pakistan)<br>***Herpestes fuscus*** (India)<br>***Herpestes javanicus*** (Pakistan)<br>***Herpestes javanicus auropunctatus*** (India)<br>***Herpestes smithii*** (India)<br>***Herpestes urva*** (India)<br>***Herpestes vitticollis*** (India)
Hyaenidae Aardwolf, hyenas			
			Hyaena hyaena (Pakistan)<br>***Proteles cristata*** (Botswana)
Mephitidae Skunks			
		Conepatus humboldtii	
Mustelidae Badgers, martens, otters, weasels, etc.			
Lutrinae Otters			
	Aonyx capensis microdon (Only the populations of Cameroon and Nigeria; all other populations are included in Appendix **II**)<br>***Aonyx cinerea***<br>***Enhydra lutris nereis***<br>***Lontra felina***<br>***Lontra longicaudis***<br>***Lontra provocax***<br>***Lutra lutra***<br>***Lutra nippon***<br>***Lutrogale perspicillata***<br>***Pteronura brasiliensis***	**Lutrinae spp.** (Except the species included in Appendix **I**)	

	Appendices I	II	III
Mustelinae Grisons, honey badgers, martens, tayra, weasels			
	Mustela nigripes		***Eira barbara*** (Honduras)<br>***Martes flavigula*** (India)<br>***Martes foina intermedia*** (India)<br>***Martes gwatkinsii*** (India)<br>***Mellivora capensis*** (Botswana)<br>***Mustela altaica*** (India)<br>***Mustela erminea ferghanae*** (India)<br>***Mustela kathiah*** (India)<br>***Mustela sibirica*** (India)
Odobenidae Walruses			
			Odobenus rosmarus (Canada)
Otariidae Fur seals, sealions			
	Arctocephalus townsendi	***Arctocephalus* spp.** (Except the species included in Appendix **I**)	
Phocidae Seals			
	***Monachus* spp.**	***Mirounga leonina***	
Procyonidae Coatis, kinkajous, olingos			
			Nasua narica (Honduras)<br>***Nasua nasua solitaria*** (Uruguay)<br>***Potos flavus*** (Honduras)
Ursidae Bears, giant pandas			
	Ailuropoda melanoleuca<br>***Helarctos malayanus***<br>***Melursus ursinus***<br>***Tremarctos ornatus***	**Ursidae spp.** (Except the species included in Appendix **I**)	

	Appendices I	II	III
	Ursus arctos (Only the populations of Bhutan, China, Mexico and Mongolia; all other populations are included in Appendix **II**)<br>***Ursus arctos isabellinus***<br>***Ursus thibetanus***		
Viverridae Binturong, civets, linsangs, otter-civet, palm civets			
	Prionodon pardicolor	***Cynogale bennettii***<br>***Hemigalus derbyanus***<br>***Prionodon linsang***	***Arctictis binturong*** (India)<br>***Civettictis civetta*** (Botswana)<br>***Paguma larvata*** (India)<br>***Paradoxurus hermaphroditus*** (India)<br>***Paradoxurus jerdoni*** (India)<br>***Viverra civettina*** (India)<br>***Viverra zibetha*** (India)<br>***Viverricula indica*** (India)
CETACEA Dolphins, porpoises, whales			
		**CETACEA spp.** (Except the species included in Appendix **I**. A zero annual export quota has been established for live specimens from the Black Sea population of *Tursiops truncatus* removed from the wild and traded for primarily commercial purposes)	
Balaenidae Bowhead whale, right whales			
	Balaena mysticetus<br>***Eubalaena* spp.**		

	Appendices I	II	III
Balaenopteridae Fin whales, humpback whales, rorquals			
	Balaenoptera acutorostrata (Except the population of West Greenland, which is included in Appendix **II**)<br>***Balaenoptera bonaerensis***<br>***Balaenoptera borealis***<br>***Balaenoptera edeni***<br>***Balaenoptera musculus***<br>***Balaenoptera omurai***<br>***Balaenoptera physalus***<br>***Megaptera novaeangliae***		
Delphinidae Dolphins			
	Orcaella brevirostris<br>***Orcaella heinsohni***<br>***Sotalia* spp.**<br>***Sousa* spp.**		
Eschrichtiidae Grey whale			
	Eschrichtius robustus		
Iniidae River dolphins			
	Lipotes vexillifer		
Neobalaenidae Pygmy right whale			
	Caperea marginata		
Phocoenidae Porpoises			
	Neophocaena asiaeorientalis<br>***Neophocaena phocaenoides***<br>***Phocoena sinus***		
Physeteridae Sperm whales			
	Physeter macrocephalus		
Platanistidae River dolphins			
	***Platanista* spp.**		
Ziphiidae Beaked whales, bottle-nosed whales			
	Berardius* spp.**<br>Hyperoodon* spp.**		

	Appendices I	II	III
CHIROPTERA			
Phyllostomidae Broad-nosed bats			
			Platyrrhinus lineatus (Uruguay)
Pteropodidae Fruit bats, flying foxes			
	Acerodon jubatus	***Acerodon* spp.** (Except the species included in Appendix I)	
	Pteropus insularis<br>***Pteropus loochoensis***<br>***Pteropus mariannus***<br>***Pteropus molossinus***<br>***Pteropus pelewensis***<br>***Pteropus pilosus***<br>***Pteropus samoensis***<br>***Pteropus tonganus***<br>***Pteropus ualanus***<br>***Pteropus yapensis***	***Pteropus* spp.** (Except the species included in Appendix I and *Pteropus brunneus*)	
CINGULATA			
Dasypodidae Armadillos			
			Cabassous tatouay (Uruguay)
		Chaetophractus nationi (A zero annual export quota has been established. All specimens shall be deemed to be specimens of species included in Appendix I and the trade in them shall be regulated accordingly)	
	Priodontes maximus		
DASYUROMORPHIA			
Dasyuridae Dunnarts			
	Sminthopsis longicaudata<br>***Sminthopsis psammophila***		

	Appendices I	II	III
DIPROTODONTIA			
Macropodidae Kangaroos, wallabies			
	Lagorchestes hirsutus<br>***Lagostrophus fasciatus***<br>***Onychogalea fraenata***	***Dendrolagus inustus***<br>***Dendrolagus ursinus***	
Phalangeridae Cuscuses			
		Phalanger intercastellanus<br>***Phalanger mimicus***<br>***Phalanger orientalis***<br>***Spilocuscus kraemeri***<br>***Spilocuscus maculatus***<br>***Spilocuscus papuensis***	
Potoroidae Rat-kangaroos			
	***Bettongia* spp.**		
Vombatidae Wombats			
	Lasiorhinus krefftii		
LAGOMORPHA			
Leporidae Hares, rabbits			
	Caprolagus hispidus<br>***Romerolagus diazi***		
MONOTREMATA			
Tachyglossidae Echidnas, spiny anteaters			
		***Zaglossus* spp.**	
PERAMELEMORPHIA			
Peramelidae Bandicoots, echymiperas			
	Perameles bougainville		
Thylacomyidae Bilbies			
	Macrotis lagotis		

	Appendices I	II	III
PERISSODACTYLA			
Equidae Horses, wild asses, zebras			
	Equus africanus (Excludes the domesticated form, which is referenced as *Equus asinus*, and is not subject to the provisions of the Convention)		
	Equus grevyi		
		Equus hemionus (Except the subspecies included in Appendix I)	
	Equus hemionus hemionus		
	Equus hemionus khur		
		Equus kiang	
	Equus przewalskii		
		Equus zebra hartmannae	
		Equus zebra zebra	
Rhinocerotidae Rhinoceroses			
	**Rhinocerotidae spp.** (Except the subspecies included in Appendix II)		
		Ceratotherium simum simum (Only the populations of Eswatini and South Africa; all other populations are included in Appendix I. For the exclusive purpose of allowing international trade in live animals to appropriate and acceptable destinations and hunting trophies. All other specimens shall be deemed to be specimens of species included in Appendix I and the trade in them shall be regulated accordingly)	
Tapiridae Tapirs			
	**Tapiridae spp.** (Except the species included in Appendix II)		
		Tapirus terrestris	

	Appendices I	II	III
PHOLIDOTA			
Manidae Pangolins			
	Manis crassicaudata<br>***Manis culionensis***<br>***Manis gigantea***<br>***Manis javanica***<br>***Manis pentadactyla***<br>***Manis temminckii***<br>***Manis tetradactyla***<br>***Manis tricuspis***	***Manis* spp.** (Except the species included in Appendix **I**)	
PILOSA			
Bradypodidae Three-toed sloths			
		Bradypus pygmaeus<br>***Bradypus variegatus***	
Myrmecophagidae American anteaters			
		Myrmecophaga tridactyla	***Tamandua mexicana*** (Guatemala)
PRIMATES Apes, monkeys			
		**PRIMATES spp.** (Except the species included in Appendix **I**)	
Atelidae Howler monkeys, spider monkeys			
	Alouatta coibensis<br>***Alouatta palliata***<br>***Alouatta pigra***<br>***Ateles geoffroyi frontatus***<br>***Ateles geoffroyi ornatus***<br>***Brachyteles arachnoides***<br>***Brachyteles hypoxanthus***<br>***Oreonax flavicauda***		

	Appendices I	II	III
Cebidae Marmosets, tamarins, new-world monkeys			
	Callimico goeldii<br>***Callithrix aurita***<br>***Callithrix flaviceps***<br>***Leontopithecus* spp.**<br>***Saguinus bicolor***<br>***Saguinus geoffroyi***<br>***Saguinus leucopus***<br>***Saguinus martinsi***<br>***Saguinus oedipus***<br>***Saimiri oerstedii***		
Cercopithecidae Old-world monkeys			
	Cercocebus galeritus<br>***Cercopithecus diana***<br>***Cercopithecus roloway***<br>***Macaca silenus***<br>***Macaca sylvanus***<br>***Mandrillus leucophaeus***<br>***Mandrillus sphinx***<br>***Nasalis larvatus***<br>***Piliocolobus kirkii***<br>***Piliocolobus rufomitratus***<br>***Presbytis potenziani***<br>***Pygathrix* spp.**<br>***Rhinopithecus* spp.**<br>***Semnopithecus ajax***<br>***Semnopithecus dussumieri***<br>***Semnopithecus entellus***<br>***Semnopithecus hector***<br>***Semnopithecus hypoleucos***<br>***Semnopithecus priam***<br>***Semnopithecus schistaceus***<br>***Simias concolor***<br>***Trachypithecus geei***		

	Appendices<br>I	II	III
	Trachypithecus pileatus<br>***Trachypithecus shortridgei***		
Cheirogaleidae Dwarf lemurs			
	**Cheirogaleidae spp.**		
Daubentoniidae Aye-aye			
	Daubentonia madagascariensis		
Hominidae Apes, chimpanzees, gorillas, orang-utans			
	Gorilla beringei<br>***Gorilla gorilla***<br>***Pan* spp.**<br>***Pongo abelii***<br>***Pongo pygmaeus***		
Hylobatidae Gibbons			
	**Hylobatidae spp.**		
Indriidae Indris, sifakas, woolly lemurs			
	**Indriidae spp.**		
Lemuridae Large lemurs			
	**Lemuridae spp.**		
Lepilemuridae Sportive lemurs			
	**Lepilemuridae spp.**		
Lorisidae Lorises			
	***Nycticebus* spp.**		
Pitheciidae Sakis, uakaris			
	Cacajao* spp.**<br>Chiropotes albinasus***		
PROBOSCIDEA			
Elephantidae Elephants			
	Elephas maximus<br>***Loxodonta africana*** (Except the populations of Botswana, Namibia, South Africa and Zimbabwe, which are included in Appendix II subject to annotation 2)		

	Appendices<br>I	II	III
		Loxodonta africana [2] (Only the populations of Botswana, Namibia, South Africa and Zimbabwe; all other populations are included in Appendix **I**)	
RODENTIA			
Chinchillidae Chinchillas			
	Chinchilla **spp.** (Specimens of the domesticated form are not subject to the provisions of the Convention)		
Cuniculidae Pacas			
			Cuniculus paca (Honduras)
Dasyproctidae Agoutis			
			Dasyprocta punctata (Honduras)

2 Populations of Botswana, Namibia, South Africa and Zimbabwe (listed in Appendix II):
For the exclusive purpose of allowing:
a) trade in hunting trophies for non-commercial purposes;
b) trade in live animals to appropriate and acceptable destinations, as defined in Resolution Conf. 11.20 (Rev. CoP18), for Botswana and Zimbabwe and for *in situ* conservation programmes for Namibia and South Africa;
c) trade in hides;
d) trade in hair;
e) trade in leather goods for commercial or non-commercial purposes for Botswana, Namibia and South Africa and for non-commercial purposes for Zimbabwe;
f) trade in individually marked and certified ekipas incorporated in finished jewellery for non-commercial purposes for Namibia and ivory carvings for non-commercial purposes for Zimbabwe;
g) trade in registered raw ivory (for Botswana, Namibia, South Africa and Zimbabwe, whole tusks and pieces) subject to the following:
   i) only registered government-owned stocks, originating in the State (excluding seized ivory and ivory of unknown origin);
   ii) only to trading partners that have been verified by the Secretariat, in consultation with the Standing Committee, to have sufficient national legislation and domestic trade controls to ensure that the imported ivory will not be re-exported and will be managed in accordance with all requirements of Resolution Conf. 10.10 (Rev. CoP18) concerning domestic manufacturing and trade;
   iii) not before the Secretariat has verified the prospective importing countries and the registered government-owned stocks;
   iv) raw ivory pursuant to the conditional sale of registered government-owned ivory stocks agreed at CoP12, which are 20,000 kg (Botswana), 10,000 kg (Namibia) and 30,000 kg (South Africa);
   v) in addition to the quantities agreed at CoP12, government-owned ivory from Botswana, Namibia, South Africa and Zimbabwe registered by 31 January 2007 and verified by the Secretariat may be traded and despatched, with the ivory in paragraph g) iv) above, in a single sale per destination under strict supervision of the Secretariat;
   vi) the proceeds of the trade are used exclusively for elephant conservation and community conservation and development programmes within or adjacent to the elephant range; and
   vii) the additional quantities specified in paragraph g) v) above shall be traded only after the Standing Committee has agreed that the above conditions have been met; and
h) no further proposals to allow trade in elephant ivory from populations already in Appendix II shall be submitted to the Conference of the Parties for the period from CoP14 and ending nine years from the date of the single sale of ivory that is to take place in accordance with provisions in paragraphs g) i), g) ii), g) iii), g) vi) and g) vii). In addition such further proposals shall be dealt with in accordance with Decisions 16.55 and 14.78 (Rev. CoP16).

On a proposal from the Secretariat, the Standing Committee can decide to cause this trade to cease partially or completely in the event of non-compliance by exporting or importing countries, or in the case of proven detrimental impacts of the trade on other elephant populations.
All other specimens shall be deemed to be specimens of species included in Appendix I and the trade in them shall be regulated accordingly.

	Appendices I	II	III
Erethizontidae New-world porcupines			
			Sphiggurus mexicanus (Honduras)<br>***Sphiggurus spinosus*** (Uruguay)
Muridae Mice, rats			
		Leporillus conditor<br>***Pseudomys fieldi***<br>***Xeromys myoides***<br>***Zyzomys pedunculatus***	
Sciuridae Ground squirrels, tree squirrels			
	Cynomys mexicanus	***Ratufa* spp.**	***Marmota caudata*** (India)<br>***Marmota himalayana*** (India)
SCANDENTIA Tree shrews			
		**SCANDENTIA spp.**	
SIRENIA			
Dugongidae Dugong			
	Dugong dugon		
Trichechidae Manatees			
	Trichechus inunguis<br>***Trichechus manatus***<br>***Trichechus senegalensis***		
**CLASS AVES (BIRDS)**			
ANSERIFORMES			
Anatidae Ducks, geese, swans, etc.			
	Anas aucklandica<br>***Anas chlorotis***<br>***Anas laysanensis***<br>***Anas nesiotis***<br>***Asarcornis scutulata***<br>***Branta canadensis leucopareia***	***Anas bernieri***<br>***Anas formosa***	

	Appendices I	II	III
		Branta ruficollis	
	Branta sandvicensis		
		Coscoroba coscoroba	
		Cygnus melancoryphus	
		Dendrocygna arborea	
			Dendrocygna autumnalis (Honduras)
			Dendrocygna bicolor (Honduras)
		Oxyura leucocephala	
	Rhodonessa caryophyllacea		
		Sarkidiornis melanotos	
APODIFORMES			
Trochilidae Hummingbirds			
		**Trochilidae spp.** (Except the species included in Appendix I)	
	Glaucis dohrnii		
CHARADRIIFORMES			
Burhinidae Thick-knees			
			Burhinus bistriatus (Guatemala)
Laridae Gulls			
	Larus relictus		
Scolopacidae Curlews, greenshanks			
	Numenius borealis<br>***Numenius tenuirostris***<br>***Tringa guttifer***		
CICONIIFORMES			
Balaenicipitidae Shoebills, whale-headed storks			
		Balaeniceps rex	
Ciconiidae Storks			
	Ciconia boyciana		
		Ciconia nigra	
	Jabiru mycteria		
	Mycteria cinerea		

	Appendices I	II	III
Phoenicopteridae Flamingos			
		**Phoenicopteridae spp.**	
Threskiornithidae Ibises, spoonbills			
		Eudocimus ruber	
		Geronticus calvus	
	Geronticus eremita		
	Nipponia nippon		
		Platalea leucorodia	
COLUMBIFORMES			
Columbidae Doves, pigeons			
	Caloenas nicobarica		
	Ducula mindorensis		
		Gallicolumba luzonica	
		***Goura* spp.**	
			Nesoenas mayeri (Mauritius)
CORACIIFORMES			
Bucerotidae Hornbills			
		***Aceros* spp.** (Except the species included in Appendix **I**)	
	Aceros nipalensis		
		***Anorrhinus* spp.**	
		***Anthracoceros* spp.**	
		***Berenicornis* spp.**	
		***Buceros* spp.** (Except the species included in Appendix **I**)	
	Buceros bicornis		
		***Penelopides* spp.**	
	Rhinoplax vigil		
		***Rhyticeros* spp.** (Except the species included in Appendix **I**)	
	Rhyticeros subruficollis		
CUCULIFORMES			
Musophagidae Turacos			
		***Tauraco* spp.**	

	Appendices I	II	III
FALCONIFORMES Eagles, falcons, hawks, vultures			
		**FALCONIFORMES spp.** (Except *Caracara lutosa* and the species of the family Cathartidae, which are not included in the Appendices; and the species included in Appendices I and III)	
Accipitridae Hawks, eagles			
	Aquila adalberti<br>***Aquila heliaca***<br>***Chondrohierax uncinatus wilsonii***<br>***Haliaeetus albicilla***<br>***Harpia harpyja***<br>***Pithecophaga jefferyi***		
Cathartidae New-world vultures			
	Gymnogyps californianus<br><br>***Vultur gryphus***		***Sarcoramphus papa*** (Honduras)
Falconidae Falcons			
	Falco araeus<br>***Falco jugger***<br>***Falco newtoni*** (Only the population of Seychelles)<br>***Falco pelegrinoides***<br>***Falco peregrinus***<br>***Falco punctatus***<br>***Falco rusticolus***		
GALLIFORMES			
Cracidae Chachalacas, currassows, guans			
	Crax blumenbachii		***Crax alberti*** (Colombia)<br><br>***Crax daubentoni*** (Colombia)<br>***Crax globulosa*** (Colombia)<br>***Crax rubra*** (Colombia, Guatemala, Honduras)

	Appendices I	II	III
	Mitu mitu		
	Oreophasis derbianus		
			Ortalis vetula (Guatemala, Honduras)
			Pauxi pauxi (Colombia)
	Penelope albipennis		
			Penelope purpurascens (Honduras)
			Penelopina nigra (Guatemala)
	Pipile jacutinga		
	Pipile pipile		
Megapodiidae Megapodes, scrubfowl			
	Macrocephalon maleo		
Phasianidae Grouse, guineafowl, partridges, peafowl, pheasants, tragopans			
		Argusianus argus	
	Catreus wallichii		
	Colinus virginianus ridgwayi		
	Crossoptilon crossoptilon		
	Crossoptilon mantchuricum		
		Gallus sonneratii	
		Ithaginis cruentus	
	Lophophorus impejanus		
	Lophophorus lhuysii		
	Lophophorus sclateri		
	Lophura edwardsi		
			Lophura leucomelanos (Pakistan)
	Lophura swinhoii		
			Meleagris ocellata (Guatemala)
			Pavo cristatus (Pakistan)
		Pavo muticus	
		Polyplectron bicalcaratum	
		Polyplectron germaini	
		Polyplectron malacense	
	Polyplectron napoleonis		
		Polyplectron schleiermacheri	

	Appendices I	II	III
			Pucrasia macrolopha (Pakistan)
	Rheinardia ocellata		
	Syrmaticus ellioti		
	Syrmaticus humiae		
	Syrmaticus mikado		
		Syrmaticus reevesii	
	Tetraogallus caspius		
	Tetraogallus tibetanus		
	Tragopan blythii		
	Tragopan caboti		
	Tragopan melanocephalus		
			Tragopan satyra (Nepal)
		Tympanuchus cupido attwateri	
GRUIFORMES			
Gruidae Cranes			
		**Gruidae spp.** (Except the species included in Appendix **I**)	
	Balearica pavonina		
	Grus americana		
	Grus canadensis nesiotes		
	Grus canadensis pulla		
	Grus japonensis		
	Grus leucogeranus		
	Grus monacha		
	Grus nigricollis		
	Grus vipio		
Otididae Bustards			
		**Otididae spp.** (Except the species included in Appendix **I**)	
	Ardeotis nigriceps		
	Chlamydotis macqueenii		
	Chlamydotis undulata		
	Houbaropsis bengalensis		
Rallidae Rails			
	Gallirallus sylvestris		

	Appendices<br>I	II	III
Rhynochetidae Kagu			
	Rhynochetos jubatus		
PASSERIFORMES			
Alaudidae Larks			
			Alauda arvensis (Population of Ukraine) (Ukraine)<br>***Galerida cristata*** (Population of Ukraine) (Ukraine)<br>***Lullula arborea*** (Population of Ukraine) (Ukraine)<br>***Melanocorypha calandra*** (Population of Ukraine) (Ukraine)
Atrichornithidae Scrub-birds			
	Atrichornis clamosus		
Cotingidae Cotingas			
	Cotinga maculata<br>***Xipholena atropurpurea***	***Rupicola* spp.**	***Cephalopterus ornatus*** (Colombia)<br>***Cephalopterus penduliger*** (Colombia)
Emberizidae Cardinals, tanagers			
		Gubernatrix cristata<br>***Paroaria capitata***<br>***Paroaria coronata***<br>***Tangara fastuosa***	***Emberiza citrinella*** (Population of Ukraine) (Ukraine)<br>***Emberiza hortulana*** (Population of Ukraine) (Ukraine)
Estrildidae Mannikins, waxbills			
		Amandava formosa<br>***Lonchura oryzivora***<br>***Poephila cincta cincta***	

	Appendices I	II	III
Fringillidae Finches			
	Carduelis cucullata	***Carduelis yarrellii***	***Carduelis cannabina*** (Population of Ukraine) (Ukraine)<br>***Carduelis carduelis*** (Population of Ukraine) (Ukraine)<br>***Carduelis flammea*** (Population of Ukraine) (Ukraine)<br>***Carduelis hornemanni*** (Population of Ukraine) (Ukraine)<br>***Carduelis spinus*** (Population of Ukraine) (Ukraine)<br>***Carpodacus erythrinus*** (Population of Ukraine) (Ukraine)<br>***Loxia curvirostra*** (Population of Ukraine) (Ukraine)<br>***Pyrrhula pyrrhula*** (Population of Ukraine) (Ukraine)<br>***Serinus serinus*** (Population of Ukraine) (Ukraine)
Hirundinidae Martins			
	Pseudochelidon sirintarae		
Icteridae New-world blackbirds			
	Xanthopsar flavus		
Meliphagidae Honeyeaters			
		Lichenostomus melanops cassidix	
Muscicapidae Old-world flycatchers			
		Cyornis ruckii<br>***Dasyornis broadbenti litoralis***<br>***Dasyornis longirostris***	***Acrocephalus rodericanus*** (Mauritius)<br>***Erithacus rubecula*** (Population of Ukraine) (Ukraine)<br>***Ficedula parva*** (Population of Ukraine) (Ukraine)

	Appendices I	II	III
		Garrulax canorus	
		Garrulax taewanus	
			Hippolais icterina (Population of Ukraine) (Ukraine)
		Leiothrix argentauris	
		Leiothrix lutea	
		Liocichla omeiensis	
			Luscinia svecica (Population of Ukraine) (Ukraine)
			Luscinia luscinia (Population of Ukraine) (Ukraine)
			Luscinia megarhynchos (Population of Ukraine) (Ukraine)
			Monticola saxatilis (Population of Ukraine) (Ukraine)
	Picathartes gymnocephalus		
	Picathartes oreas		
			Sylvia atricapilla (Population of Ukraine) (Ukraine)
			Sylvia borin (Population of Ukraine) (Ukraine)
			Sylvia curruca (Population of Ukraine) (Ukraine)
			Sylvia nisoria (Population of Ukraine) (Ukraine)
			Terpsiphone bourbonnensis (Mauritius)
			Turdus merula (Population of Ukraine) (Ukraine)
			Turdus philomelos (Population of Ukraine) (Ukraine)
Oriolidae Orioles			
			Oriolus oriolus (Population of Ukraine) (Ukraine)
Paradisaeidae Birds of paradise			
		**Paradisaeidae spp.**	

	Appendices<br>I	 II	 III
Paridae Tits			
			Parus ater (Population of Ukraine) (Ukraine)
Pittidae Pittas			
	Pitta gurneyi<br>***Pitta kochi***	***Pitta guajana***<br>***Pitta nympha***	
Pycnonotidae Bulbuls			
		Pycnonotus zeylanicus	
Sturnidae Mynas, starlings			
	Leucopsar rothschildi	***Gracula religiosa***	
Troglodytidae Wrens			
			Troglodytes troglodytes (Population of Ukraine) (Ukraine)
Zosteropidae White-eyes			
	Zosterops albogularis		
PELECANIFORMES			
Fregatidae Frigatebirds			
	Fregata andrewsi		
Pelecanidae Pelicans			
	Pelecanus crispus		
Sulidae Gannets			
	Papasula abbotti		
PICIFORMES			
Capitonidae Barbets			
			Semnornis ramphastinus (Colombia)
Picidae Woodpeckers			
	Dryocopus javensis richardsi		
Ramphastidae Toucans			
		Pteroglossus aracari	***Baillonius bailloni*** (Argentina)<br>***Pteroglossus castanotis*** (Argentina)

	Appendices<br>I	 II	 III
		Pteroglossus viridis	
			Ramphastos dicolorus (Argentina)
		Ramphastos sulfuratus<br>***Ramphastos toco***<br>***Ramphastos tucanus***<br>***Ramphastos vitellinus***	
			Selenidera maculirostris (Argentina)
PODICIPEDIFORMES			
Podicipedidae Grebes			
	Podilymbus gigas		
PROCELLARIIFORMES			
Diomedeidae Albatrosses			
	Phoebastria albatrus		
PSITTACIFORMES			
		**PSITTACIFORMES spp.** (Except the species included in Appendix I and *Agapornis roseicollis, Melopsittacus undulatus, Nymphicus hollandicus* and *Psittacula krameri*, which are not included in the Appendices)	
Cacatuidae Cockatoos			
	Cacatua goffiniana<br>***Cacatua haematuropygia***<br>***Cacatua moluccensis***<br>***Cacatua sulphurea***<br>***Probosciger aterrimus***		
Loriidae Lories, lorikeets			
	Eos histrio<br>***Vini ultramarina***		
Psittacidae Amazons, macaws, parakeets, parrots			
	Amazona arausiaca<br>***Amazona auropalliata***<br>***Amazona barbadensis***<br>***Amazona brasiliensis***		

	Appendices I	II	III
	Amazona finschi<br>*Amazona guildingii*<br>*Amazona imperialis*<br>*Amazona leucocephala*<br>*Amazona oratrix*<br>*Amazona pretrei*<br>*Amazona rhodocorytha*<br>*Amazona tucumana*<br>*Amazona versicolor*<br>*Amazona vinacea*<br>*Amazona viridigenalis*<br>*Amazona vittata*<br>*Anodorhynchus* spp.<br>*Ara ambiguus*<br>*Ara glaucogularis*<br>*Ara macao*<br>*Ara militaris*<br>*Ara rubrogenys*<br>*Cyanopsitta spixii*<br>*Cyanoramphus cookii*<br>*Cyanoramphus forbesi*<br>*Cyanoramphus novaezelandiae*<br>*Cyanoramphus saisseti*<br>*Cyclopsitta diophthalma coxeni*<br>*Eunymphicus cornutus*<br>*Guarouba guarouba*<br>*Neophema chrysogaster*<br>*Ognorhynchus icterotis*<br>*Pezoporus occidentalis*<br>*Pezoporus wallicus*<br>*Pionopsitta pileata*<br>*Primolius couloni*<br>*Primolius maracana*<br>*Psephotus chrysopterygius*<br>*Psephotus dissimilis*		

	Appendices I	II	III
	Psephotus pulcherrimus<br>***Psittacula echo***<br>***Psittacus erithacus***<br>***Pyrrhura cruentata***<br>***Rhynchopsitta* spp.**<br>***Strigops habroptilus***		
RHEIFORMES			
Rheidae Rheas			
	Pterocnemia pennata (Except *Pterocnemia pennata pennata* which is included in Appendix II)	***Pterocnemia pennata pennata***<br>***Rhea americana***	
SPHENISCIFORMES			
Spheniscidae Penguins			
	Spheniscus humboldti	***Spheniscus demersus***	
STRIGIFORMES Owls			
		**STRIGIFORMES spp.** (Except the species included in Appendix I and *Sceloglaux albifacies*)	
Strigidae Owls			
	Heteroglaux blewitti<br>***Mimizuku gurneyi***<br>***Ninox natalis***		
Tytonidae Barn owls			
	Tyto soumagnei		
STRUTHIONIFORMES			
Struthionidae Ostriches			
	Struthio camelus (Only the populations of Algeria, Burkina Faso, Cameroon, the Central African Republic, Chad, Mali, Mauritania, Morocco, the Niger, Nigeria, Senegal and the Sudan; all other populations are not included in the Appendices)		

	Appendices I	II	III
TINAMIFORMES			
Tinamidae Tinamous			
	Tinamus solitarius		
TROGONIFORMES			
Trogonidae Quetzals			
	Pharomachrus mocinno		
**CLASS REPTILIA (REPTILES)**			
CROCODYLIA Alligators, caimans, crocodiles			
		**CROCODYLIA spp.** (Except the species included in Appendix **I**)	
Alligatoridae Alligators, caimans			
	Alligator sinensis<br>***Caiman crocodilus apaporiensis***<br>***Caiman latirostris*** (Except the population of Argentina, which is included in Appendix **II**)<br>***Melanosuchus niger*** (Except the population of Brazil, which is included in Appendix **II**, and the population of Ecuador, which is included in Appendix **II** and is subject to a zero annual export quota until an annual export quota has been approved by the CITES Secretariat and the IUCN/SSC Crocodile Specialist Group)		

	Appendices<br>I	II	III
Crocodylidae Crocodiles			
	Crocodylus acutus (Except the population of the Integrated Management District of Mangroves of the Bay of Cispata, Tinajones, La Balsa and Surrounding Areas, Department of Córdoba, Colombia, and the population of Cuba, which are included in Appendix II; and the population of Mexico, which is included in Appendix II and is subject to a zero export quota for wild specimens for commercial purposes)<br>***Crocodylus cataphractus***<br>***Crocodylus intermedius***<br>***Crocodylus mindorensis***<br>***Crocodylus moreletii*** (Except the population of Belize, which is included in Appendix II with a zero quota for wild specimens traded for commercial purposes, and the population of Mexico, which is included in Appendix II)<br>***Crocodylus niloticus*** [Except the populations of Botswana, Egypt (subject to a zero quota for wild specimens traded for commercial purposes), Ethiopia, Kenya, Madagascar, Malawi, Mozambique, Namibia, South Africa, Uganda, the United Republic of Tanzania (subject to an annual export quota of no more than 1,600 wild specimens including hunting trophies, in addition to ranched specimens), Zambia and Zimbabwe, which are included in Appendix II]<br>***Crocodylus palustris***		

	Appendices I	II	III
	Crocodylus porosus {Except the populations of Australia, Indonesia, Malaysia [wild harvest restricted to the State of Sarawak and a zero quota for wild specimens for the other States of Malaysia (Sabah and Peninsular Malaysia), with no change in the zero quota unless approved by the Parties] and Papua New Guinea, which are included in Appendix II}<br>***Crocodylus rhombifer***<br>***Crocodylus siamensis***<br>***Osteolaemus tetraspis***<br>***Tomistoma schlegelii***		
Gavialidae Gavials			
	Gavialis gangeticus		
RHYNCHOCEPHALIA			
Sphenodontidae Tuataras			
	***Sphenodon* spp.**		
SAURIA			
Agamidae Spiny-tailed lizards, agamas			
			Calotes ceylonensis (Sri Lanka)<br>***Calotes desilvai*** (Sri Lanka)<br>***Calotes liocephalus*** (Sri Lanka)<br>***Calotes liolepis*** (Sri Lanka)<br>***Calotes manamendrai*** (Sri Lanka)<br>***Calotes nigrilabris*** (Sri Lanka)<br>***Calotes pethiyagodai*** (Sri Lanka)
		Ceratophora aspera (Zero export quota for wild specimens for commercial purposes)	
	Ceratophora erdeleni<br>***Ceratophora karu***		
		Ceratophora stoddartii (Zero export quota for wild specimens for commercial purposes)	

	Appendices		
	I	II	III
	Ceratophora tennentii		
	Cophotis ceylanica		
	Cophotis dumbara		
			***Ctenophorus* spp.** (Australia)
			***Intellagama* spp.** (Australia)
		Lyriocephalus scutatus (Zero export quota for wild specimens for commercial purposes)	
		***Saara* spp.**	
			***Tympanocryptis* spp.** (Australia)
		***Uromastyx* spp.**	
Anguidae Alligator lizards			
		***Abronia* spp.** [Except the species included in Appendix **I** (zero export quota for wild specimens for *Abronia aurita, A. gaiophantasma, A. montecristoi, A. salvadorensis* and *A. vasconcelosii*)]	
	Abronia anzuetoi		
	Abronia campbelli		
	Abronia fimbriata		
	Abronia frosti		
	Abronia meledona		
Chamaeleonidae Chameleons			
		***Archaius* spp.**	
		***Bradypodion* spp.**	
		***Brookesia* spp.** (Except the species included in Appendix **I**)	
	Brookesia perarmata		
		***Calumma* spp.**	
		***Chamaeleo* spp.**	
		***Furcifer* spp.**	
		***Kinyongia* spp.**	
		***Nadzikambia* spp.**	
		***Palleon* spp.**	
		***Rhampholeon* spp.**	

	Appendices I	II	III
		***Rieppeleon* spp.**	
		***Trioceros* spp.**	
Cordylidae Spiny-tailed lizards			
		***Cordylus* spp.**	
		***Hemicordylus* spp.**	
		***Karusaurus* spp.**	
		***Namazonurus* spp.**	
		***Ninurta* spp.**	
		***Ouroborus* spp.**	
		***Pseudocordylus* spp.**	
		***Smaug* spp.**	
Eublepharidae Eyelid geckos			
		***Goniurosaurus* spp.** (Except the species native to Japan)	
			Goniurosaurus kuroiwae[#18] (Japan)
			Goniurosaurus orientalis[#18] (Japan)
			Goniurosaurus sengokui[#18] (Japan)
			Goniurosaurus splendens[#18] (Japan)
			Goniurosaurus toyamai[#18] (Japan)
			Goniurosaurus yamashinae[#18] (Japan)
Gekkonidae Geckos			
			***Carphodactylus* spp.** (Australia)
	Cnemaspis psychedelica		
			***Dactylocnemis* spp.** (New Zealand)
		Gekko gecko	
	Gonatodes daudini		
			***Hoplodactylus* spp.** (New Zealand)
	Lygodactylus williamsi		
			***Mokopirirakau* spp.** (New Zealand)
		Nactus serpensinsula	
		***Naultinus* spp.**	
			***Nephrurus* spp.** (Australia)
			***Orraya* spp.** (Australia)

[#18] Excluding parts and derivatives, other than eggs.

	Appendices<br>I	 II	 III
		Paroedura androyensis	
		Paroedura masobe	
		***Phelsuma* spp.**	
			***Phyllurus* spp.** (Australia)
		***Rhoptropella* spp.**	
			***Saltuarius* spp.** (Australia)
			Sphaerodactylus armasi (Cuba)
			Sphaerodactylus celicara (Cuba)
			Sphaerodactylus dimorphicus (Cuba)
			Sphaerodactylus intermedius (Cuba)
			Sphaerodactylus nigropunctatus alayoi (Cuba)
			Sphaerodactylus nigropunctatus granti (Cuba)
			Sphaerodactylus nigropunctatus lissodesmus (Cuba)
			Sphaerodactylus nigropunctatus ocujal (Cuba)
			Sphaerodactylus nigropunctatus strategus (Cuba)
			Sphaerodactylus notatus atactus (Cuba)
			Sphaerodactylus oliveri (Cuba)
			Sphaerodactylus pimienta (Cuba)
			Sphaerodactylus ruibali (Cuba)
			Sphaerodactylus siboney (Cuba)
			Sphaerodactylus torrei (Cuba)
			***Strophurus* spp.** (Australia)
			***Toropuku* spp.** (New Zealand)
			***Tukutuku* spp.** (New Zealand)
			***Underwoodisaurus* spp.** (Australia)
		***Uroplatus* spp.**	
			***Uvidicolus* spp.** (Australia)
			***Woodworthia* spp.** (New Zealand)

	Appendices I	II	III
Helodermatidae Beaded lizards, Gila monsters			
	Heloderma horridum charlesbogerti	***Heloderma* spp.** (Except the subspecies included in Appendix I)	
Iguanidae Iguanas			
		Amblyrhynchus cristatus	
	***Brachylophus* spp.**		
		***Conolophus* spp.**	
		***Ctenosaura* spp.**	
	***Cyclura* spp.**		
		***Iguana* spp.**	
		Phrynosoma blainvillii	
		Phrynosoma cerroense	
		Phrynosoma coronatum	
		Phrynosoma wigginsi	
	Sauromalus varius		
Lacertidae Lizards			
	Gallotia simonyi		
		Podarcis lilfordi	
		Podarcis pityusensis	
Lanthanotidae Earless monitor lizards			
		**Lanthanotidae spp.** (Zero export quota for wild specimens for commercial purposes)	
Polychrotidae Anoles			
			Anolis agueroi (Cuba)
			Anolis baracoae (Cuba)
			Anolis barbatus (Cuba)
			Anolis chamaeleonides (Cuba)
			Anolis equestris (Cuba)
			Anolis guamuhaya (Cuba)
			Anolis luteogularis (Cuba)
			Anolis pigmaequestris (Cuba)
			Anolis porcus (Cuba)

	Appendices I	II	III
Scincidae Skinks			
		Corucia zebrata	***Egernia* spp.** (Australia)<br>***Tiliqua adelaidensis*** (Australia)<br>***Tiliqua multifasciata*** (Australia)<br>***Tiliqua nigrolutea*** (Australia)<br>***Tiliqua occipitalis*** (Australia)<br>***Tiliqua rugosa*** (Australia)<br>***Tiliqua scincoides intermedia*** (Australia)<br>***Tiliqua scincoides scincoides*** (Australia)
Teiidae Caiman lizards, tegu lizards			
		Crocodilurus amazonicus<br>***Dracaena* spp.**<br>***Salvator* spp.**<br>***Tupinambis* spp.**	
Varanidae Monitor lizards			
	Varanus bengalensis<br>***Varanus flavescens***<br>***Varanus griseus***<br>***Varanus komodoensis***<br>***Varanus nebulosus***	***Varanus* spp.** (Except the species included in Appendix **I**)	
Xenosauridae Chinese crocodile lizard			
	Shinisaurus crocodilurus		
SERPENTES			
Boidae Boas			
	Acrantophis* spp.**<br>Boa constrictor occidentalis***	**Boidae spp.** (Except the species included in Appendix **I**)	

	Appendices I	II	III
	Epicrates inornatus<br>***Epicrates monensis***<br>***Epicrates subflavus***<br>***Sanzinia madagascariensis***		
Bolyeriidae Round Island boas			
	Bolyeria multocarinata<br>***Casarea dussumieri***	**Bolyeriidae spp.** (Except the species included in Appendix **I**)	
Colubridae Typical snakes, water snakes, whipsnakes			
		Clelia clelia<br>***Cyclagras gigas***<br>***Elachistodon westermanni***<br>***Ptyas mucosus***	***Atretium schistosum*** (India)<br>***Cerberus rynchops*** (India)<br>***Xenochrophis piscator*** (India)<br>***Xenochrophis schnurrenbergeri*** (India)<br>***Xenochrophis tytleri*** (India)
Elapidae Cobras, coral snakes			
		Hoplocephalus bungaroides<br>***Naja atra***<br>***Naja kaouthia***<br>***Naja mandalayensis***<br>***Naja naja***<br>***Naja oxiana***<br>***Naja philippinensis***<br>***Naja sagittifera***<br>***Naja samarensis***<br>***Naja siamensis***	***Micrurus diastema*** (Honduras)<br>***Micrurus nigrocinctus*** (Honduras)<br>***Micrurus ruatanus*** (Honduras)

	Appendices I	II	III
		Naja sputatrix<br>***Naja sumatrana***<br>***Ophiophagus hannah***	
Loxocemidae Mexican dwarf boas			
		**Loxocemidae spp.**	
Pythonidae Pythons			
	Python molurus molurus	**Pythonidae spp.** (Except the subspecies included in Appendix **I**)	
Tropidophiidae Wood boas			
		**Tropidophiidae spp.**	
Viperidae Vipers			
	Vipera ursinii (Only the population of Europe, except the area which formerly constituted the Union of Soviet Socialist Republics; these latter populations are not included in the Appendices)	***Atheris desaixi***<br>***Bitis worthingtoni***<br>***Pseudocerastes urarachnoides***<br>***Trimeresurus mangshanensis***<br>***Vipera wagneri***	***Crotalus durissus*** (Honduras)<br>***Daboia russelii*** (India)
TESTUDINES			
Carettochelyidae Pig-nosed turtles			
		Carettochelys insculpta	
Chelidae Austro-American sideneck turtles			
	Pseudemydura umbrina	***Chelodina mccordi*** (Zero export quota for specimens from the wild)	
Cheloniidae Sea turtles			
	**Cheloniidae spp.**		
Chelydridae Snapping turtles			

	Appendices I	II	III
			Chelydra serpentina (United States of America)<br>***Macrochelys temminckii*** (United States of America)
Dermatemydidae Central American river turtles			
		Dermatemys mawii	
Dermochelyidae Leatherback turtles			
	Dermochelys coriacea		
Emydidae Box turtles, freshwater turtles			
		Clemmys guttata	
		Emydoidea blandingii	
			Emys orbicularis (Population of Ukraine) (Ukraine)
		Glyptemys insculpta	
	Glyptemys muhlenbergii		
			***Graptemys* spp.** (United States of America)
		Malaclemys terrapin	
		***Terrapene* spp.** (Except the species included in Appendix **I**)	
	Terrapene coahuila		
Geoemydidae Box turtles, freshwater turtles			
	Batagur affinis		
	Batagur baska		
		Batagur borneoensis (Zero quota for wild specimens for commercial purposes)	
		Batagur dhongoka	
		Batagur kachuga	
		Batagur trivittata (Zero quota for wild specimens for commercial purposes)	

	Appendices I	II	III
		***Cuora* spp.** (Except the species included in Appendix **I**; zero quota for wild specimens for commercial purposes for *Cuora aurocapitata, C. flavomarginata, C. galbinifrons, C. mccordi, C. mouhotii, C. pani, C. trifasciata, C. yunnanensis and C. zhoui*)	
	Cuora bourreti		
	Cuora picturata		
		***Cyclemys* spp.**	
	Geoclemys hamiltonii		
		Geoemyda japonica	
		Geoemyda spengleri	
		Hardella thurjii	
		Heosemys annandalii (Zero quota for wild specimens for commercial purposes)	
		Heosemys depressa (Zero quota for wild specimens for commercial purposes)	
		Heosemys grandis	
		Heosemys spinosa	
		Leucocephalon yuwonoi	
		Malayemys macrocephala	
		Malayemys subtrijuga	
	Mauremys annamensis		
			Mauremys iversoni (China)
		Mauremys japonica	
			Mauremys megalocephala (China)
		Mauremys mutica	
		Mauremys nigricans	
			Mauremys pritchardi (China)
			Mauremys reevesii (China)
			Mauremys sinensis (China)
	Melanochelys tricarinata		

	Appendices I	II	III
		Melanochelys trijuga	
	Morenia ocellata		
		Morenia petersi	
		Notochelys platynota	
			Ocadia glyphistoma (China)
			Ocadia philippeni (China)
		Orlitia borneensis (Zero quota for wild specimens for commercial purposes)	
		***Pangshura* spp.** (Except the species included in Appendix **I**)	
	Pangshura tecta		
		Sacalia bealei	
			Sacalia pseudocellata (China)
		Sacalia quadriocellata	
		Siebenrockiella crassicollis	
		Siebenrockiella leytensis	
		Vijayachelys silvatica	
Platysternidae Big-headed turtles			
	**Platysternidae spp.**		
Podocnemididae Afro-American sideneck turtles			
		Erymnochelys madagascariensis	
		Peltocephalus dumerilianus	
		***Podocnemis* spp.**	
Testudinidae Tortoises			
		**Testudinidae spp.** (Except the species included in Appendix **I**. A zero annual export quota has been established for *Centrochelys sulcata* for specimens removed from the wild and traded for primarily commercial purposes)	
	Astrochelys radiata		
	Astrochelys yniphora		
	Chelonoidis niger		
	Geochelone elegans		
	Geochelone platynota		

	Appendices I	II	III
	Gopherus flavomarginatus<br>***Malacochersus tornieri***<br>***Psammobates geometricus***<br>***Pyxis arachnoides***<br>***Pyxis planicauda***<br>***Testudo kleinmanni***		
Trionychidae Softshell turtles			
		Amyda cartilaginea	
			Apalone ferox (United States of America)
			Apalone mutica (United States of America)
			Apalone spinifera (Except the subspecies included in Appendix **I**) (United States of America)
	Apalone spinifera atra		
		***Chitra* spp.** (Except the species included in Appendix **I**)	
	Chitra chitra<br>***Chitra vandijki***		
		Cyclanorbis elegans<br>***Cyclanorbis senegalensis***<br>***Cycloderma aubryi***<br>***Cycloderma frenatum***<br>***Dogania subplana***<br>***Lissemys ceylonensis***<br>***Lissemys punctata***<br>***Lissemys scutata***<br>***Nilssonia formosa***	
	Nilssonia gangetica<br>***Nilssonia hurum***		
		Nilssonia leithii	
	Nilssonia nigricans		
		Palea steindachneri<br>***Pelochelys* spp.**	

	Appendices I	Appendices II	Appendices III
		Pelodiscus axenaria<br>***Pelodiscus maackii***<br>***Pelodiscus parviformis***<br>***Rafetus euphraticus***<br>***Rafetus swinhoei***<br>***Trionyx triunguis***	
**CLASS AMPHIBIA (AMPHIBIANS)**			
ANURA			
Aromobatidae Cryptic forest frogs			
		Allobates femoralis<br>***Allobates hodli***<br>***Allobates myersi***<br>***Allobates zaparo***<br>***Anomaloglossus rufulus***	
Bufonidae Toads			
	Altiphrynoides* spp.**<br>Amietophrynus channingi***<br>***Amietophrynus superciliaris***<br>***Atelopus zeteki***<br>***Incilius periglenes***<br>***Nectophrynoides* spp.**<br>***Nimbaphrynoides* spp.**		
Calyptocephalellidae Chilean toads			
			Calyptocephalella gayi (Chile)
Dendrobatidae Poison frogs			
		Adelphobates* spp.**<br>Ameerega* spp.**<br>***Andinobates* spp.**<br>***Dendrobates* spp.**<br>***Epipedobates* spp.**<br>***Excidobates* spp.**<br>***Hyloxalus azureiventris***<br>***Minyobates* spp.**<br>***Oophaga* spp.**	

	Appendices I	II	III
		Phyllobates* spp.**<br>Ranitomeya* spp.**	
Dicroglossidae Frogs			
		Euphlyctis hexadactylus<br>***Hoplobatrachus tigerinus***	
Hylidae Tree frogs			
		***Agalychnis* spp.**	
Mantellidae Mantella frogs			
		***Mantella* spp.**	
Microhylidae Tomato frogs			
		Dyscophus antongilii<br>***Dyscophus guineti***<br>***Dyscophus insularis***<br>***Scaphiophryne boribory***<br>***Scaphiophryne gottlebei***<br>***Scaphiophryne marmorata***<br>***Scaphiophryne spinosa***	
Myobatrachidae Gastric-brooding frogs			
		***Rheobatrachus* spp.** (Except *Rheobatrachus silus* and *Rheobatrachus vitellinus* which are not included in the Appendices)	
Telmatobiidae Andean water frogs			
	Telmatobius culeus		
CAUDATA			
Ambystomatidae Axolotls, mole salamanders			
		Ambystoma dumerilii<br>***Ambystoma mexicanum***	
Cryptobranchidae Giant salamanders			
	***Andrias* spp.**		***Cryptobranchus alleganiensis*** (United States of America)

	Appendices I	II	III
Hynobiidae Asiatic salamanders			
			Hynobius amjiensis (China)
Salamandridae Newts and salamanders			
	Neurergus kaiseri	***Echinotriton chinhaiensis***<br>***Echinotriton maxiquadratus***<br>***Paramesotriton* spp.**<br>***Tylototriton* spp.**	***Echinotriton andersoni*** #18 (Japan)<br>***Salamandra algira*** (Algeria)
**CLASS ELASMOBRANCHII (SHARKS)**			
CARCHARHINIFORMES			
Carcharhinidae Requiem sharks			
		Carcharhinus falciformis<br>***Carcharhinus longimanus***	
Sphyrnidae Hammerhead sharks			
		Sphyrna lewini<br>***Sphyrna mokarran***<br>***Sphyrna zygaena***	
LAMNIFORMES			
Alopiidae Thresher sharks			
		***Alopias* spp.**	
Cetorhinidae Basking sharks			
		Cetorhinus maximus	
Lamnidae Mackerel sharks			
		Carcharodon carcharias<br>***Isurus oxyrinchus***<br>***Isurus paucus***<br>***Lamna nasus***	
MYLIOBATIFORMES			

#18 Excluding parts and derivatives, other than eggs.

	Appendices I	II	III
Myliobatidae Eagle and mobulid rays			
		Manta* spp.**<br>Mobula* spp.**	
Potamotrygonidae Freshwater stingrays			
			Paratrygon aiereba (Colombia)<br>***Potamotrygon* spp.** (population of Brazil) (Brazil)<br>***Potamotrygon constellata*** (Colombia)<br>***Potamotrygon magdalenae*** (Colombia)<br>***Potamotrygon motoro*** (Colombia)<br>***Potamotrygon orbignyi*** (Colombia)<br>***Potamotrygon schroederi*** (Colombia)<br>***Potamotrygon scobina*** (Colombia)<br>***Potamotrygon yepezi*** (Colombia)
ORECTOLOBIFORMES			
Rhincodontidae Whale sharks			
		Rhincodon typus	
PRISTIFORMES			
Pristidae Sawfishes			
	**Pristidae spp.**		
RHINOPRISTIFORMES			
Glaucostegidae Guitarfishes			
		***Glaucostegus* spp.**	
Rhinidae Wedgefishes			
		**Rhinidae spp.**	
**CLASS ACTINOPTERI (FISHES)**			
ACIPENSERIFORMES			
		**ACIPENSERIFORMES spp.** (Except the species included in Appendix I)	
Acipenseridae Sturgeons			
	Acipenser brevirostrum<br>***Acipenser sturio***		

	Appendices I	II	III
ANGUILLIFORMES			
Anguillidae Freshwater eels			
		Anguilla anguilla	
CYPRINIFORMES			
Catostomidae Cui-ui			
	Chasmistes cujus		
Cyprinidae Carps			
	Probarbus jullieni	***Caecobarbus geertsii***	
OSTEOGLOSSIFORMES			
Arapaimidae Arapaimas			
		Arapaima gigas	
Osteoglossidae Bonytongue			
	Scleropages formosus<br>***Scleropages inscriptus***		
PERCIFORMES			
Labridae Wrasses			
		Cheilinus undulatus	
Pomacanthidae Angelfishes			
		Holacanthus clarionensis	***Holacanthus limbaughi*** (France)
Sciaenidae Totoaba			
	Totoaba macdonaldi		
SILURIFORMES			
Loricariidae Armoured catfishes			
			Hypancistrus zebra (Brazil)
Pangasiidae Pangasid catfish			
	Pangasianodon gigas		
SYNGNATHIFORMES			
Syngnathidae Pipefishes, seahorses			
		***Hippocampus* spp.**	

	I	II	III
**CLASS DIPNEUSTI (LUNGFISHES)**			
CERATODONTIFORMES			
Neoceratodontidae Australian lungfishes			
		Neoceratodus forsteri	
**CLASS COELACANTHI (COELACANTHS)**			
COELACANTHIFORMES			
Latimeriidae Coelacanths			
	***Latimeria* spp.**		
**PHYLUM ECHINODERMATA CLASS HOLOTHUROIDEA (SEA CUCUMBERS)**			
ASPIDOCHIROTIDA			
Stichopodidae Sea cucumbers			
			Isostichopus fuscus (Ecuador)
HOLOTHURIIDA			
Holothuriidae Teatfishes, Sea cucumbers			
		Holothuria fuscogilva<br>***Holothuria nobilis***<br>***Holothuria whitmaei***	
**PHYLUM ARTHROPODA CLASS ARACHNIDA (SCORPIONS AND SPIDERS)**			
ARANEAE			
Theraphosidae Red-kneed tarantulas, tarantulas			
		Aphonopelma albiceps<br>***Aphonopelma pallidum***<br>***Brachypelma* spp.**<br>***Poecilotheria* spp.**	
SCORPIONES			
Scorpionidae Scorpions			

	Appendices I	II	III
		Pandinus camerounensis<br>*Pandinus dictator*<br>*Pandinus gambiensis*<br>*Pandinus imperator*<br>*Pandinus roeseli*	

	Appendices I	II	III
**CLASS INSECTA (INSECTS)**			
COLEOPTERA			
Lucanidae Stag beetles			
			***Colophon* spp.** (South Africa)
Scarabaeidae Scarab beetles			
		Dynastes satanas	
LEPIDOPTERA			
Nymphalidae Brush-footed butterflies			
			Agrias amydon boliviensis (Plurinational State of Bolivia)<br>***Morpho godartii lachaumei*** (Plurinational State of Bolivia)<br>***Prepona praeneste buckleyana*** (Plurinational State of Bolivia)
Papilionidae Birdwing butterflies, swallowtail butterflies			
	Achillides chikae chikae		
	Achillides chikae hermeli		
		Atrophaneura jophon	
		Atrophaneura pandiyana	
		***Bhutanitis* spp.**	
		***Ornithoptera* spp.** (Except the species included in Appendix I)	
	Ornithoptera alexandrae		
	Papilio homerus		
		Papilio hospiton	
	Parides burchellanus		
		Parnassius apollo	
		***Teinopalpus* spp.**	
		***Trogonoptera* spp.**	
		***Troides* spp.**	

	Appendices I	II	III
**PHYLUM ANNELIDA CLASS HIRUDINOIDEA (LEECHES)**			
ARHYNCHOBDELLIDA			
Hirudinidae Medicinal leeches			
		Hirudo medicinalis<br>***Hirudo verbana***	
**PHYLUM MOLLUSCA CLASS BIVALVIA (CLAMS AND MUSSELS)**			
MYTILOIDA			
Mytilidae Marine mussels			
		Lithophaga lithophaga	
UNIONOIDA			
Unionidae Freshwater mussels, pearly mussels			
	Conradilla caelata		
		Cyprogenia aberti	
	Dromus dromas<br>***Epioblasma curtisi***<br>***Epioblasma florentina***<br>***Epioblasma sampsonii***<br>***Epioblasma sulcata perobliqua***<br>***Epioblasma torulosa gubernaculum***		
		Epioblasma torulosa rangiana	
	Epioblasma torulosa torulosa<br>***Epioblasma turgidula***<br>***Epioblasma walkeri***<br>***Fusconaia cuneolus***<br>***Fusconaia edgariana***<br>***Lampsilis higginsii***<br>***Lampsilis orbiculata orbiculata***<br>***Lampsilis satur***<br>***Lampsilis virescens***<br>***Plethobasus cicatricosus***<br>***Plethobasus cooperianus***		

	Appendices I	II	III
	Pleurobema plenum<br>***Potamilus capax***<br>***Quadrula intermedia***<br>***Quadrula sparsa***<br>***Toxolasma cylindrella***<br>***Unio nickliniana***<br>***Unio tampicoensis tecomatensis***<br>***Villosa trabalis***	***Pleurobema clava***	
VENEROIDA			
Tridacnidae Giant clams			
		**Tridacnidae spp.**	
**CLASS CEPHALOPODA (SQUIDS, OCTOPUSES, CUTTLEFISH)**			
NAUTILIDA			
Nautilidae Chambered nautilus			
		**Nautilidae spp.**	
**CLASS GASTROPODA (SNAILS AND CONCHES)**			
MESOGASTROPODA			
Strombidae True conchs			
		Strombus gigas	
STYLOMMATOPHORA			
Achatinellidae Agate snails, oahu tree snails			
	***Achatinella* spp.**		
Camaenidae Green tree snails			
		Papustyla pulcherrima	
Cepolidae Helicoid terrestrial snails			
	***Polymita* spp.**		

	Appendices I	II	III
**PHYLUM CNIDARIA**<br>**CLASS ANTHOZOA (CORALS AND SEA ANEMONES)**			
ANTIPATHARIA Black corals			
		**ANTIPATHARIA spp.**	
GORGONACEAE			
Coralliidae Red and pink corals			
			Corallium elatius (China)<br>***Corallium japonicum*** (China)<br>***Corallium konjoi*** (China)<br>***Corallium secundum*** (China)
HELIOPORACEA			
Helioporidae Blue corals			
		**Helioporidae spp.** (Includes only the species *Heliopora coerulea*. Fossils are not subject to the provisions of the Convention)	
SCLERACTINIA Stony corals			
		**SCLERACTINIA spp.** (Fossils are not subject to the provisions of the Convention)	
STOLONIFERA			
Tubiporidae Organ-pipe corals			
		**Tubiporidae spp.** (Fossils are not subject to the provisions of the Convention)	

	Appendices I	II	III
**CLASS HYDROZOA (SEA FERNS, FIRE CORALS AND STINGING MEDUSAE)**			
MILLEPORINA			
Milleporidae Fire corals			
		**Milleporidae spp.** (Fossils are not subject to the provisions of the Convention)	
STYLASTERINA			
Stylasteridae Lace corals			
		**Stylasteridae spp.** (Fossils are not subject to the provisions of the Convention)	

	Appendices I	II	III
**FLORA (PLANTS)**			
AGAVACEAE Agaves			
	Agave parviflora	***Agave victoriae-reginae*** #4<br>***Nolina interrata***<br>***Yucca queretaroensis***	
AMARYLLIDACEAE Snowdrops, sternbergias			
		Galanthus* spp.** #4<br>Sternbergia* spp.** #4	
ANACARDIACEAE Cashews			
		Operculicarya decaryi<br>***Operculicarya hyphaenoides***<br>***Operculicarya pachypus***	
APOCYNACEAE Elephant trunks, hoodias			
	Pachypodium ambongense<br>***Pachypodium baronii***<br>***Pachypodium decaryi***	***Hoodia* spp.** #9<br>***Pachypodium* spp.** #4 (Except the species included in Appendix I)	

#4 All parts and derivatives, except:

a) seeds (including seedpods of Orchidaceae), spores and pollen (including pollinia). The exemption does not apply to seeds from Cactaceae spp. exported from Mexico, and to seeds from *Beccariophoenix madagascariensis* and *Dypsis decaryi* exported from Madagascar;
b) seedling or tissue cultures obtained *in vitro*, in solid or liquid media, transported in sterile containers;
c) cut flowers of artificially propagated plants;
d) fruits, and parts and derivatives thereof, of naturalized or artificially propagated plants of the genus *Vanilla* (Orchidaceae) and of the family Cactaceae;
e) stems, flowers, and parts and derivatives thereof, of naturalized or artificially propagated plants of the genera *Opuntia* subgenus *Opuntia* and *Selenicereus* (Cactaceae); and
f) finished products of *Aloe ferox* and *Euphorbia antisyphilitica* packaged and ready for retail trade.

#9 All parts and derivatives except those bearing a label:
"Produced from *Hoodia* spp. material obtained through controlled harvesting and production under the terms of an agreement with the relevant CITES Management Authority of [Botswana under agreement No. BW/xxxxxx] [Namibia under agreement No. NA/xxxxxx] [South Africa under agreement No. ZA/xxxxxx]".

	Appendices I	II	III
		Rauvolfia serpentina #2	
ARALIACEAE Ginseng			
		Panax ginseng #3 (Only the population of the Russian Federation; no other population is included in the Appendices)<br>***Panax quinquefolius*** #3	
ARAUCARIACEAE Monkey-puzzle trees			
	Araucaria araucana		
ASPARAGACEAE Includes ponytail palms			
		***Beaucarnea* spp.**	
BERBERIDACEAE May-apple			
		Podophyllum hexandrum #2	
BROMELIACEAE Air plants, bromelias			
		Tillandsia harrisii #4<br>***Tillandsia kammii*** #4<br>***Tillandsia xerographica*** #4	

#2 All parts and derivatives except:
a) seeds and pollen; and
b) finished products packaged and ready for retail trade.

#3 Whole and sliced roots and parts of roots, excluding manufactured parts or derivatives, such as powders, pills, extracts, tonics, teas and confectionery.

#4 All parts and derivatives, except:
a) seeds (including seedpods of Orchidaceae), spores and pollen (including pollinia). The exemption does not apply to seeds from Cactaceae spp. exported from Mexico, and to seeds from *Beccariophoenix madagascariensis* and *Dypsis decaryi* exported from Madagascar;
b) seedling or tissue cultures obtained *in vitro*, in solid or liquid media, transported in sterile containers;
c) cut flowers of artificially propagated plants;
d) fruits, and parts and derivatives thereof, of naturalized or artificially propagated plants of the genus *Vanilla* (Orchidaceae) and of the family Cactaceae;
e) stems, flowers, and parts and derivatives thereof, of naturalized or artificially propagated plants of the genera *Opuntia* subgenus *Opuntia* and *Selenicereus* (Cactaceae); and
f) finished products of *Aloe ferox* and *Euphorbia antisyphilitica* packaged and ready for retail trade.

	Appendices I	II	III
CACTACEAE Cacti			
		**CACTACEAE spp.**[9] [#4] (Except the species included in Appendix I and except *Pereskia* spp., *Pereskiopsis* spp. and *Quiabentia* spp.)	
	***Ariocarpus* spp.**		
	Astrophytum asterias		
	Aztekium ritteri		
	Coryphantha werdermannii		
	***Discocactus* spp.**		
	Echinocereus ferreiranus* ssp. *lindsayorum		
	Echinocereus schmollii		
	Escobaria minima		
	Escobaria sneedii		
	Mammillaria pectinifera (includes **ssp. *solisioides***)		
	Melocactus conoideus		
	Melocactus deinacanthus		
	Melocactus glaucescens		
	Melocactus paucispinus		
	Obregonia denegrii		

[9] Artificially propagated specimens of the following hybrids and/or cultivars are not subject to the provisions of the Convention:
– *Hatiora* x *graeseri*
– *Schlumbergera* x *buckleyi*
– *Schlumbergera russelliana* x *Schlumbergera truncata*
– *Schlumbergera orssichiana* x *Schlumbergera truncata*
– *Schlumbergera opuntioides* x *Schlumbergera truncata*
– *Schlumbergera truncata* (cultivars)
– Cactaceae spp. colour mutants grafted on the following grafting stocks: *Harrisia* 'Jusbertii', *Hylocereus trigonus* or *Hylocereus undatus*
– *Opuntia microdasys* (cultivars).

[#4] All parts and derivatives, except:
a) seeds (including seedpods of Orchidaceae), spores and pollen (including pollinia). The exemption does not apply to seeds from Cactaceae spp. exported from Mexico, and to seeds from *Beccariophoenix madagascariensis* and *Dypsis decaryi* exported from Madagascar;
b) seedling or tissue cultures obtained *in vitro*, in solid or liquid media, transported in sterile containers;
c) cut flowers of artificially propagated plants;
d) fruits, and parts and derivatives thereof, of naturalized or artificially propagated plants of the genus *Vanilla* (Orchidaceae) and of the family Cactaceae;
e) stems, flowers, and parts and derivatives thereof, of naturalized or artificially propagated plants of the genera *Opuntia* subgenus *Opuntia* and *Selenicereus* (Cactaceae); and
f) finished products of *Aloe ferox* and *Euphorbia antisyphilitica* packaged and ready for retail trade.

	Appendices<br>I	 II	 III
	Pachycereus militaris<br>***Pediocactus bradyi***<br>***Pediocactus knowltonii***<br>***Pediocactus paradinei***<br>***Pediocactus peeblesianus***<br>***Pediocactus sileri***<br>***Pelecyphora* spp.**<br>***Sclerocactus blainei***<br>***Sclerocactus brevihamatus* ssp. *tobuschii***<br>***Sclerocactus brevispinus***<br>***Sclerocactus cloverae***<br>***Sclerocactus erectocentrus***<br>***Sclerocactus glaucus***<br>***Sclerocactus mariposensis***<br>***Sclerocactus mesae-verdae***<br>***Sclerocactus nyensis***<br>***Sclerocactus papyracanthus***<br>***Sclerocactus pubispinus***<br>***Sclerocactus sileri***<br>***Sclerocactus wetlandicus***<br>***Sclerocactus wrightiae***<br>***Strombocactus* spp.**<br>***Turbinicarpus* spp.**<br>***Uebelmannia* spp.**		

	Appendices I	II	III
CARYOCARACEAE Ajo			
		Caryocar costaricense #4	
COMPOSITAE (Asteraceae) Kuth			
	Saussurea costus		
CUCURBITACEAE Melons, gourds, cucurbits			
		Zygosicyos pubescens<br>***Zygosicyos tripartitus***	
CUPRESSACEAE Alerce, cypresses			
	Fitzroya cupressoides<br>***Pilgerodendron uviferum***	***Widdringtonia whytei***	
CYATHEACEAE Tree-ferns			
		***Cyathea* spp.** #4	
CYCADACEAE Cycads			
	Cycas beddomei	**CYCADACEAE spp.** #4 (Except the species included in Appendix **I**)	
DICKSONIACEAE Tree-ferns			
		Cibotium barometz #4<br>***Dicksonia* spp.** #4 (Only the populations of the Americas; no other population is included in the Appendices)	
DIDIEREACEAE Alluaudias, didiereas			
		**DIDIEREACEAE spp.** #4	

#4 All parts and derivatives, except:

a) seeds (including seedpods of Orchidaceae), spores and pollen (including pollinia). The exemption does not apply to seeds from Cactaceae spp. exported from Mexico, and to seeds from *Beccariophoenix madagascariensis* and *Dypsis decaryi* exported from Madagascar;
b) seedling or tissue cultures obtained *in vitro*, in solid or liquid media, transported in sterile containers;
c) cut flowers of artificially propagated plants;
d) fruits, and parts and derivatives thereof, of naturalized or artificially propagated plants of the genus *Vanilla* (Orchidaceae) and of the family Cactaceae;
e) stems, flowers, and parts and derivatives thereof, of naturalized or artificially propagated plants of the genera *Opuntia* subgenus *Opuntia* and *Selenicereus* (Cactaceae); and
f) finished products of *Aloe ferox* and *Euphorbia antisyphilitica* packaged and ready for retail trade.

	I	Appendices<br>II	III
DIOSCOREACEAE Elephant's foot, kniss			
		Dioscorea deltoidea #4	
DROSERACEAE Venus' flytrap			
		Dionaea muscipula #4	
EBENACEAE Ebonies			
		***Diospyros* spp.** #5 (Populations of Madagascar)	
EUPHORBIACEAE Spurges			
		***Euphorbia* spp.** #4 (Succulent species only except *Euphorbia misera* and the species included in Appendix I. Artificially propagated specimens of cultivars of *Euphorbia trigona*, artificially propagated specimens of crested, fan-shaped or colour mutants of *Euphorbia lactea*, when grafted on artificially propagated root stock of *Euphorbia neriifolia*, and artificially propagated specimens of cultivars of *Euphorbia* 'Milii' when they are traded in shipments of 100 or more plants and readily recognizable as artificially propagated specimens, are not subject to the provisions of the Convention)	

#4 All parts and derivatives, except:

a) seeds (including seedpods of Orchidaceae), spores and pollen (including pollinia). The exemption does not apply to seeds from Cactaceae spp. exported from Mexico, and to seeds from *Beccariophoenix madagascariensis* and *Dypsis decaryi* exported from Madagascar;
b) seedling or tissue cultures obtained *in vitro*, in solid or liquid media, transported in sterile containers;
c) cut flowers of artificially propagated plants;
d) fruits, and parts and derivatives thereof, of naturalized or artificially propagated plants of the genus *Vanilla* (Orchidaceae) and of the family Cactaceae;
e) stems, flowers, and parts and derivatives thereof, of naturalized or artificially propagated plants of the genera *Opuntia* subgenus *Opuntia* and *Selenicereus* (Cactaceae); and
f) finished products of *Aloe ferox* and *Euphorbia antisyphilitica* packaged and ready for retail trade.

#5 Logs, sawn wood and veneer sheets.

	Appendices I	II	III
	Euphorbia ambovombensis<br>***Euphorbia capsaintemariensis***<br>***Euphorbia cremersii*** (Includes the *forma viridifolia* and the var. *rakotozafyi*)<br>***Euphorbia cylindrifolia*** (Includes the ssp. *tuberifera*)<br>***Euphorbia decaryi*** (Includes the vars. *ampanihyensis, robinsonii* and *spirosticha*)<br>***Euphorbia francoisii***<br>***Euphorbia moratii*** (Includes the vars. *antsingiensis*, *bemarahensis* and *multiflora*)<br>***Euphorbia parvicyathophora***<br>***Euphorbia quartziticola***<br>***Euphorbia tulearensis***		
FAGACEAE Beeches			
			Quercus mongolica #5 (Russian Federation)
FOUQUIERIACEAE Ocotillos			
	Fouquieria fasciculata<br>***Fouquieria purpusii***	***Fouquieria columnaris*** #4	

#5 Logs, sawn wood and veneer sheets.

#4 All parts and derivatives, except:

a) seeds (including seedpods of Orchidaceae), spores and pollen (including pollinia). The exemption does not apply to seeds from Cactaceae spp. exported from Mexico, and to seeds from *Beccariophoenix madagascariensis* and *Dypsis decaryi* exported from Madagascar;
b) seedling or tissue cultures obtained *in vitro*, in solid or liquid media, transported in sterile containers;
c) cut flowers of artificially propagated plants;
d) fruits, and parts and derivatives thereof, of naturalized or artificially propagated plants of the genus *Vanilla* (Orchidaceae) and of the family Cactaceae;
e) stems, flowers, and parts and derivatives thereof, of naturalized or artificially propagated plants of the genera *Opuntia* subgenus *Opuntia* and *Selenicereus* (Cactaceae); and
f) finished products of *Aloe ferox* and *Euphorbia antisyphilitica* packaged and ready for retail trade.

	Appendices I	II	III
GNETACEAE Gnetums			
			Gnetum montanum #1 (Nepal)
JUGLANDACEAE Gavilan			
		Oreomunnea pterocarpa #4	
LAURACEAE Laurels			
		Aniba rosaeodora #12	
LEGUMINOSAE (Fabaceae) Afrormosia, cristobal, palisander, rosewood, sandalwood			
	Dalbergia nigra	***Dalbergia* spp.** #15 (except for the species listed in Appendix I)	
			Dipteryx panamensis (Costa Rica, Nicaragua)
		Guibourtia demeusei #15	
		Guibourtia pellegriniana #15	
		Guibourtia tessmannii #15	
		Paubrasilia echinata #10	

#1 All parts and derivatives, except:
a) seeds, spores and pollen (including pollinia);
b) seedling or tissue cultures obtained *in vitro*, in solid or liquid media, transported in sterile containers;
c) cut flowers of artificially propagated plants; and
d) fruits, and parts and derivatives thereof, of artificially propagated plants of the genus *Vanilla*.

#4 All parts and derivatives, except:
a) seeds (including seedpods of Orchidaceae), spores and pollen (including pollinia). The exemption does not apply to seeds from Cactaceae spp. exported from Mexico, and to seeds from *Beccariophoenix madagascariensis* and *Dypsis decaryi* exported from Madagascar;
b) seedling or tissue cultures obtained *in vitro*, in solid or liquid media, transported in sterile containers;
c) cut flowers of artificially propagated plants;
d) fruits, and parts and derivatives thereof, of naturalized or artificially propagated plants of the genus *Vanilla* (Orchidaceae) and of the family Cactaceae;
e) stems, flowers, and parts and derivatives thereof, of naturalized or artificially propagated plants of the genera *Opuntia* subgenus *Opuntia* and *Selenicereus* (Cactaceae); and
f) finished products of *Aloe ferox* and *Euphorbia antisyphilitica* packaged and ready for retail trade.

#12 Logs, sawn wood, veneer sheets, plywood and extracts. Finished products containing such extracts as ingredients, including fragrances, are not considered to be covered by this annotation.

#15 All parts and derivatives, except:
a) Leaves, flowers, pollen, fruits, and seeds;
b) Finished products to a maximum weight of wood of the listed species of up to 10 kg per shipment;
c) Finished musical instruments, finished musical instrument parts and finished musical instrument accessories;
d) Parts and derivatives of *Dalbergia cochinchinensis*, which are covered by Annotation # 4; and
e) Parts and derivatives of *Dalbergia* spp. originating and exported from Mexico, which are covered by Annotation # 6.

#10 Logs, sawn wood, veneer sheets, including unfinished wood articles used for the fabrication of bows for stringed musical instruments.

	Appendices I	II	III
		Pericopsis elata #17<br>***Platymiscium parviflorum*** #4<br>***Pterocarpus erinaceus***<br>***Pterocarpus santalinus*** #7<br>***Pterocarpus tinctorius*** #6<br>***Senna meridionalis***	
LILIACEAE Aloes			
	Aloe albida<br>***Aloe albiflora***<br>***Aloe alfredii***<br>***Aloe bakeri***<br>***Aloe bellatula***<br>***Aloe calcairophila***<br>***Aloe compressa*** (Includes the vars. *paucituberculata, rugosquamosa* and *schistophila*)<br>***Aloe delphinensis***<br>***Aloe descoingsii***<br>***Aloe fragilis***<br>***Aloe haworthioides*** (Includes the var. *aurantiaca*)	***Aloe* spp.** #4 (Except the species included in Appendix I. Also excludes *Aloe vera*, also referenced as *Aloe barbadensis* which is not included in the Appendices)	

#17 Logs, sawn wood, veneer sheets, plywood and transformed wood.

#4 All parts and derivatives, except:

a) seeds (including seedpods of Orchidaceae), spores and pollen (including pollinia). The exemption does not apply to seeds from Cactaceae spp. exported from Mexico, and to seeds from *Beccariophoenix madagascariensis* and *Dypsis decaryi* exported from Madagascar;
b) seedling or tissue cultures obtained *in vitro*, in solid or liquid media, transported in sterile containers;
c) cut flowers of artificially propagated plants;
d) fruits, and parts and derivatives thereof, of naturalized or artificially propagated plants of the genus *Vanilla* (Orchidaceae) and of the family Cactaceae;
e) stems, flowers, and parts and derivatives thereof, of naturalized or artificially propagated plants of the genera *Opuntia* subgenus *Opuntia* and *Selenicereus* (Cactaceae); and
f) finished products of *Aloe ferox* and *Euphorbia antisyphilitica* packaged and ready for retail trade.

#7 Logs, woodchips, powder and extracts.

#6 Logs, sawn wood, veneer sheets and plywood.

	Appendices I	II	III
	Aloe helenae<br>***Aloe laeta*** (Includes the var. *maniaensis*)<br>***Aloe parallelifolia***<br>***Aloe parvula***<br>***Aloe pillansii***<br>***Aloe polyphylla***<br>***Aloe rauhii***<br>***Aloe suzannae***<br>***Aloe versicolor***<br>***Aloe vossii***		
MAGNOLIACEAE Magnolias			
			Magnolia liliifera* var. *obovata #1 (Nepal)
MALVACEAE Includes baobabs			
		Adansonia grandidieri #16	
MELIACEAE Mahoganies, West Indian cedar			
		***Cedrela* spp.** #6 (Populations of the Neotropics)	

#1 All parts and derivatives, except:
a) seeds, spores and pollen (including pollinia);
b) seedling or tissue cultures obtained *in vitro*, in solid or liquid media, transported in sterile containers;
c) cut flowers of artificially propagated plants; and
d) fruits, and parts and derivatives thereof, of artificially propagated plants of the genus *Vanilla*.

#16 Seeds, fruits and oils.

#6 Logs, sawn wood, veneer sheets and plywood.

	Appendices<br>I	II	III
		Swietenia humilis [#4]<br>***Swietenia macrophylla*** [#6] (Populations of the Neotropics)<br>***Swietenia mahagoni*** [#5]	
NEPENTHACEAE Pitcher-plants (Old World)			
	Nepenthes khasiana<br>***Nepenthes rajah***	***Nepenthes* spp.** [#4] (Except the species included in Appendix **I**)	
OLEACEAE Ashes, etc.			
			Fraxinus mandshurica [#5] (Russian Federation)
ORCHIDACEAE Orchids			
		**ORCHIDACEAE spp.** [10] [#4]<br>(Except the species included in Appendix I)	

[#4] All parts and derivatives, except:

a) seeds (including seedpods of Orchidaceae), spores and pollen (including pollinia). The exemption does not apply to seeds from Cactaceae spp. exported from Mexico, and to seeds from *Beccariophoenix madagascariensis* and *Dypsis decaryi* exported from Madagascar;
b) seedling or tissue cultures obtained *in vitro*, in solid or liquid media, transported in sterile containers;
c) cut flowers of artificially propagated plants;
d) fruits, and parts and derivatives thereof, of naturalized or artificially propagated plants of the genus *Vanilla* (Orchidaceae) and of the family Cactaceae;
e) stems, flowers, and parts and derivatives thereof, of naturalized or artificially propagated plants of the genera *Opuntia* subgenus *Opuntia* and *Selenicereus* (Cactaceae); and
f) finished products of *Aloe ferox* and *Euphorbia antisyphilitica* packaged and ready for retail trade.

[#6] Logs, sawn wood, veneer sheets and plywood.

[#5] Logs, sawn wood and veneer sheets.

[10] Artificially propagated hybrids of the following genera are not subject to the provisions of the Convention, if conditions, as indicated under a) and b), are met: *Cymbidium, Dendrobium, Phalaenopsis* and *Vanda*:

a) Specimens are readily recognizable as artificially propagated and do not show any signs of having been collected in the wild such as mechanical damage or strong dehydration resulting from collection, irregular growth and heterogeneous size and shape within a taxon and shipment, algae or other epiphyllous organisms adhering to leaves, or damage by insects or other pests; and
b) i) when shipped in non-flowering state, the specimens must be traded in shipments consisting of individual containers (such as cartons, boxes, crates or individual shelves of CC-containers) each containing 20 or more plants of the same hybrid; the plants within each container must exhibit a high degree of uniformity and healthiness; and the shipment must be accompanied by documentation, such as an invoice, which clearly states the number of plants of each hybrid; or
   ii) when shipped in flowering state, with at least one fully open flower per specimen, no minimum number of specimens per shipment is required but specimens must be professionally processed for commercial retail sale, e.g. labelled with printed labels or packaged with printed packages indicating the name of the hybrid and the country of final processing. This should be clearly visible and allow easy verification.

Plants not clearly qualifying for the exemption must be accompanied by appropriate CITES documents.

	Appendices I	II	III
	(For all of the following Appendix-I species, seedling or tissue cultures obtained *in vitro*, in solid or liquid media, and transported in sterile containers are not subject to the provisions of the Convention only if the specimens meet the definition of 'artificially propagated' agreed by the Conference of the Parties)<br>***Aerangis ellisii***<br>***Cattleya jongheana***<br>***Cattleya lobata***<br>***Dendrobium cruentum***<br>***Mexipedium xerophyticum***<br>***Paphiopedilum* spp.**<br>***Peristeria elata***<br>***Phragmipedium* spp.**<br>***Renanthera imschootiana***		
OROBANCHACEAE Broomrapes			
		Cistanche deserticola #4	
PALMAE (Arecaceae) Palms			
	Dypsis decipiens	***Beccariophoenix madagascariensis*** #4<br>***Dypsis decaryi*** #4<br>***Lemurophoenix halleuxii***<br>***Marojejya darianii***<br>***Ravenea louvelii***	***Lodoicea maldivica*** #13 (Seychelles)

#4 All parts and derivatives, except:

a) seeds (including seedpods of Orchidaceae), spores and pollen (including pollinia). The exemption does not apply to seeds from Cactaceae spp. exported from Mexico, and to seeds from *Beccariophoenix madagascariensis* and *Dypsis decaryi* exported from Madagascar;
b) seedling or tissue cultures obtained *in vitro*, in solid or liquid media, transported in sterile containers;
c) cut flowers of artificially propagated plants;
d) fruits, and parts and derivatives thereof, of naturalized or artificially propagated plants of the genus *Vanilla* (Orchidaceae) and of the family Cactaceae;
e) stems, flowers, and parts and derivatives thereof, of naturalized or artificially propagated plants of the genera *Opuntia* subgenus *Opuntia* and *Selenicereus* (Cactaceae); and
f) finished products of *Aloe ferox* and *Euphorbia antisyphilitica* packaged and ready for retail trade.

#13 The kernel (also known as 'endosperm', 'pulp' or 'copra') and any derivative thereof, except finished products packaged and ready for retail trade.

	Appendices I	II	III
		Ravenea rivularis<br>***Satranala decussilvae***<br>***Voanioala gerardii***	
PAPAVERACEAE Poppy			
			Meconopsis regia #1 (Nepal)
PASSIFLORACEAE Passion-flowers			
		Adenia firingalavensis<br>***Adenia olaboensis***<br>***Adenia subsessilifolia***	
PEDALIACEAE Sesames			
		Uncarina grandidieri<br>***Uncarina stellulifera***	
PINACEAE Firs and pines			
	Abies guatemalensis		***Pinus koraiensis*** #5 (Russian Federation)
PODOCARPACEAE Podocarps			
	Podocarpus parlatorei		***Podocarpus neriifolius*** #1 (Nepal)

#1 All parts and derivatives, except:
a) seeds, spores and pollen (including pollinia);
b) seedling or tissue cultures obtained *in vitro*, in solid or liquid media, transported in sterile containers;
c) cut flowers of artificially propagated plants; and
d) fruits, and parts and derivatives thereof, of artificially propagated plants of the genus *Vanilla*.

#5 Logs, sawn wood and veneer sheets.

	Appendices I	II	III
PORTULACACEAE Lewisias, portulacas, purslanes			
		Anacampseros* spp.** #4<br>Avonia* spp.** #4<br>***Lewisia serrata*** #4	
PRIMULACEAE Cyclamens			
		***Cyclamen* spp.** 11 #4	
RANUNCULACEAE Golden seals, yellow adonis, yellow root			
		Adonis vernalis #2<br>***Hydrastis canadensis*** #8	
ROSACEAE African cherry, stinkwood			
		Prunus africana #4	
RUBIACEAE Ayugue			
	Balmea stormiae		
SANTALACEAE Sandalwoods			
		Osyris lanceolata #2 (Populations of Burundi, Ethiopia, Kenya, Rwanda, Uganda and the United Republic of Tanzania)	

#4 All parts and derivatives, except:
a) seeds (including seedpods of Orchidaceae), spores and pollen (including pollinia). The exemption does not apply to seeds from Cactaceae spp. exported from Mexico, and to seeds from *Beccariophoenix madagascariensis* and *Dypsis decaryi* exported from Madagascar;
b) seedling or tissue cultures obtained *in vitro*, in solid or liquid media, transported in sterile containers;
c) cut flowers of artificially propagated plants;
d) fruits, and parts and derivatives thereof, of naturalized or artificially propagated plants of the genus *Vanilla* (Orchidaceae) and of the family Cactaceae;
e) stems, flowers, and parts and derivatives thereof, of naturalized or artificially propagated plants of the genera *Opuntia* subgenus *Opuntia* and *Selenicereus* (Cactaceae); and
f) finished products of *Aloe ferox* and *Euphorbia antisyphilitica* packaged and ready for retail trade.

11 Artificially propagated specimens of cultivars of *Cyclamen persicum* are not subject to the provisions of the Convention. However, the exemption does not apply to such specimens traded as dormant tubers.

#2 All parts and derivatives except:
a) seeds and pollen; and
b) finished products packaged and ready for retail trade.

#8 Underground parts (i.e. roots, rhizomes): whole, parts and powdered.

	Appendices I	II	III
SARRACENIACEAE Pitcher-plants (New World)			
	Sarracenia oreophila<br>***Sarracenia rubra* ssp. *alabamensis***<br>***Sarracenia rubra* ssp. *jonesii***	***Sarracenia* spp.** #4 (Except the species included in Appendix I)	
SCROPHULARIACEAE Kutki			
		Picrorhiza kurrooa #2 (Excludes *Picrorhiza scrophulariiflora)*	
STANGERIACEAE Stangerias			
	Stangeria eriopus	***Bowenia* spp.** #4	
TAXACEAE Himalayan yew			
		Taxus chinensis and infraspecific taxa of this species #2<br>***Taxus cuspidata*** and infraspecific taxa of this species [12] #2<br>***Taxus fuana*** and infraspecific taxa of this species #2<br>***Taxus sumatrana*** and infraspecific taxa of this species #2<br>***Taxus wallichiana*** #2	
THYMELAEACEAE (Aquilariaceae) Agarwood, ramin			

#4 All parts and derivatives, except:
a) seeds (including seedpods of Orchidaceae), spores and pollen (including pollinia). The exemption does not apply to seeds from Cactaceae spp. exported from Mexico, and to seeds from *Beccariophoenix madagascariensis* and *Dypsis decaryi* exported from Madagascar;
b) seedling or tissue cultures obtained *in vitro*, in solid or liquid media, transported in sterile containers;
c) cut flowers of artificially propagated plants;
d) fruits, and parts and derivatives thereof, of naturalized or artificially propagated plants of the genus *Vanilla* (Orchidaceae) and of the family Cactaceae;
e) stems, flowers, and parts and derivatives thereof, of naturalized or artificially propagated plants of the genera *Opuntia* subgenus *Opuntia* and *Selenicereus* (Cactaceae); and
f) finished products of *Aloe ferox* and *Euphorbia antisyphilitica* packaged and ready for retail trade.

#2 All parts and derivatives except:
a) seeds and pollen; and
b) finished products packaged and ready for retail trade.

12 Artificially propagated hybrids and cultivars of *Taxus cuspidata,* live, in pots or other small containers, each consignment being accompanied by a label or document stating the name of the taxon or taxa and the text 'artificially propagated', are not subject to the provisions of the Convention.

	Appendices I	II	III
		Aquilaria* spp.** #14<br>Gonystylus* spp.** #4<br>***Gyrinops* spp.** #14	
TROCHODENDRACEAE (Tetracentraceae) Tetracentron			
			Tetracentron sinense #1 (Nepal)
VALERIANACEAE Himalayan spikenard			
		Nardostachys grandiflora #2	
VITACEAE Grapes			
		Cyphostemma elephantopus<br>***Cyphostemma laza***<br>***Cyphostemma montagnacii***	

#14 All parts and derivatives except:
a) seeds and pollen;
b) seedling or tissue cultures obtained *in vitro*, in solid or liquid media, transported in sterile containers;
c) fruits;
d) leaves;
e) exhausted agarwood powder, including compressed powder in all shapes; and
f) finished products packaged and ready for retail trade, this exemption does not apply to wood chips, beads, prayer beads and carvings.

#4 All parts and derivatives, except:
a) seeds (including seedpods of Orchidaceae), spores and pollen (including pollinia). The exemption does not apply to seeds from Cactaceae spp. exported from Mexico, and to seeds from *Beccariophoenix madagascariensis* and *Dypsis decaryi* exported from Madagascar;
b) seedling or tissue cultures obtained *in vitro*, in solid or liquid media, transported in sterile containers;
c) cut flowers of artificially propagated plants;
d) fruits, and parts and derivatives thereof, of naturalized or artificially propagated plants of the genus *Vanilla* (Orchidaceae) and of the family Cactaceae;
e) stems, flowers, and parts and derivatives thereof, of naturalized or artificially propagated plants of the genera *Opuntia* subgenus *Opuntia* and *Selenicereus* (Cactaceae); and
f) finished products of *Aloe ferox* and *Euphorbia antisyphilitica* packaged and ready for retail trade.

#1 All parts and derivatives, except:
a) seeds, spores and pollen (including pollinia);
b) seedling or tissue cultures obtained *in vitro*, in solid or liquid media, transported in sterile containers;
c) cut flowers of artificially propagated plants; and
d) fruits, and parts and derivatives thereof, of artificially propagated plants of the genus *Vanilla*.

#2 All parts and derivatives except:
a) seeds and pollen; and
b) finished products packaged and ready for retail trade.

	Appendices<br>I	 II	 III
WELWITSCHIACEAE Welwitschia			
		Welwitschia mirabilis #4	
ZAMIACEAE Cycads			
	Ceratozamia* spp.**<br>Encephalartos* spp.**<br>***Microcycas calocoma***<br>***Zamia restrepoi***	**ZAMIACEAE spp.** #4 (Except the species included in Appendix **I**)	
ZINGIBERACEAE Ginger lily, Natal ginger			
		Hedychium philippinense #4<br>***Siphonochilus aethiopicus*** (Populations of Mozambique, South Africa, Eswatini and Zimbabwe)	
ZYGOPHYLLACEAE Lignum-vitae			
		Bulnesia sarmientoi #11<br>***Guaiacum* spp.** #2	

#4 All parts and derivatives, except:
a) seeds (including seedpods of Orchidaceae), spores and pollen (including pollinia). The exemption does not apply to seeds from Cactaceae spp. exported from Mexico, and to seeds from *Beccariophoenix madagascariensis* and *Dypsis decaryi* exported from Madagascar;
b) seedling or tissue cultures obtained *in vitro*, in solid or liquid media, transported in sterile containers;
c) cut flowers of artificially propagated plants;
d) fruits, and parts and derivatives thereof, of naturalized or artificially propagated plants of the genus *Vanilla* (Orchidaceae) and of the family Cactaceae;
e) stems, flowers, and parts and derivatives thereof, of naturalized or artificially propagated plants of the genera *Opuntia* subgenus *Opuntia* and *Selenicereus* (Cactaceae); and
f) finished products of *Aloe ferox* and *Euphorbia antisyphilitica* packaged and ready for retail trade.

#11 Logs, sawn wood, veneer sheets, plywood, powder and extracts. Finished products containing such extracts as ingredients, including fragrances, are not considered to be covered by this annotation.

#2 All parts and derivatives except:
a) seeds and pollen; and
b) finished products packaged and ready for retail trade.
