# Supplementary material for: Nocturnal surveys of lined seahorses reveal increased densities and seasonal recruitment patterns
Source: Ecol Evol. 2023 Jan 11;13(1):e9573. doi: 10.1002/ece3.9573 (PMC9834011; doi:10.1002/ece3.9573)
Supplement: Supplementary file 2 — Figure S1. [file ECE3-13-e9573-s002.pdf]

Supplemental Figure 1: Hotspot figures created using abundances of females (S1a), males (S1b) and juveniles (S1c). To determine aggregations of seahorses by sex and abundance, a hot spot analysis (Getis-Ord Gi) was conducted in ArcMap (version 10.7). Seahorse data points were separated by sex with only nighttime data included since it was the only sampling time where the seahorses were able to be measured against a standardized grid and fish reliably identified by size. Season again was not separated out due to the smaller sample size in the wet season.

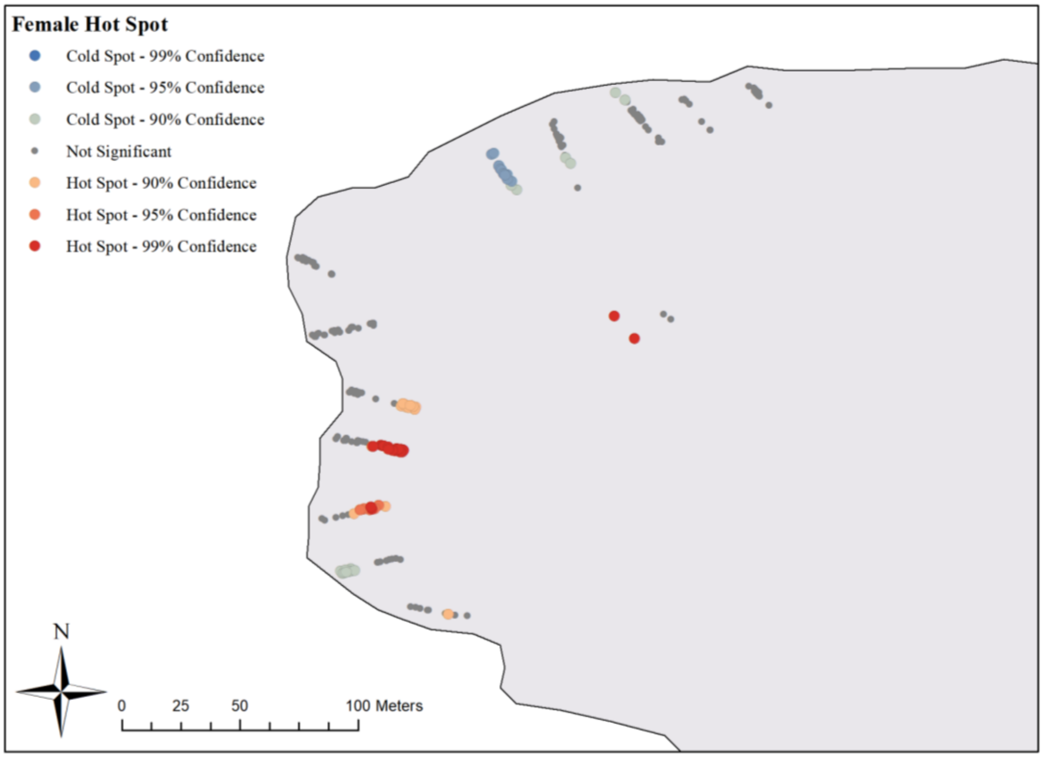

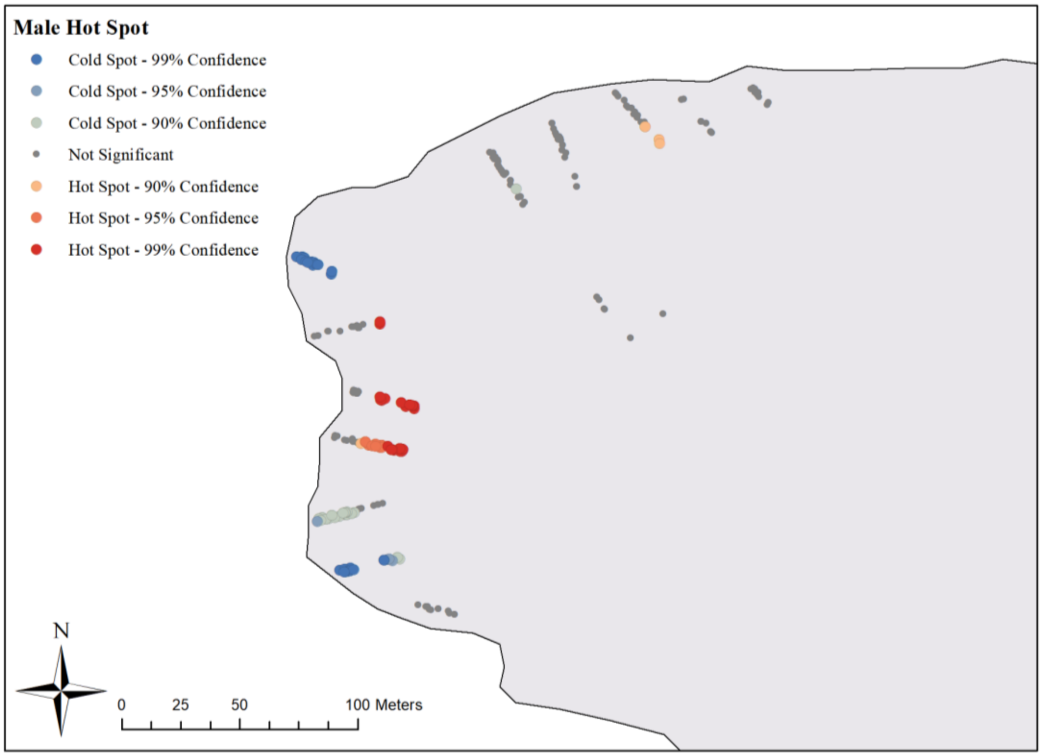
Male Hot Spot
Cold Spot - 99% Confidence
Cold Spot - 95% Confidence
Cold Spot - 90% Confidence
Not Significant
Hot Spot - 90% Confidence
Hot Spot - 95% Confidence
Hot Spot - 99% Confidence
N
0
25
50
100 Meters

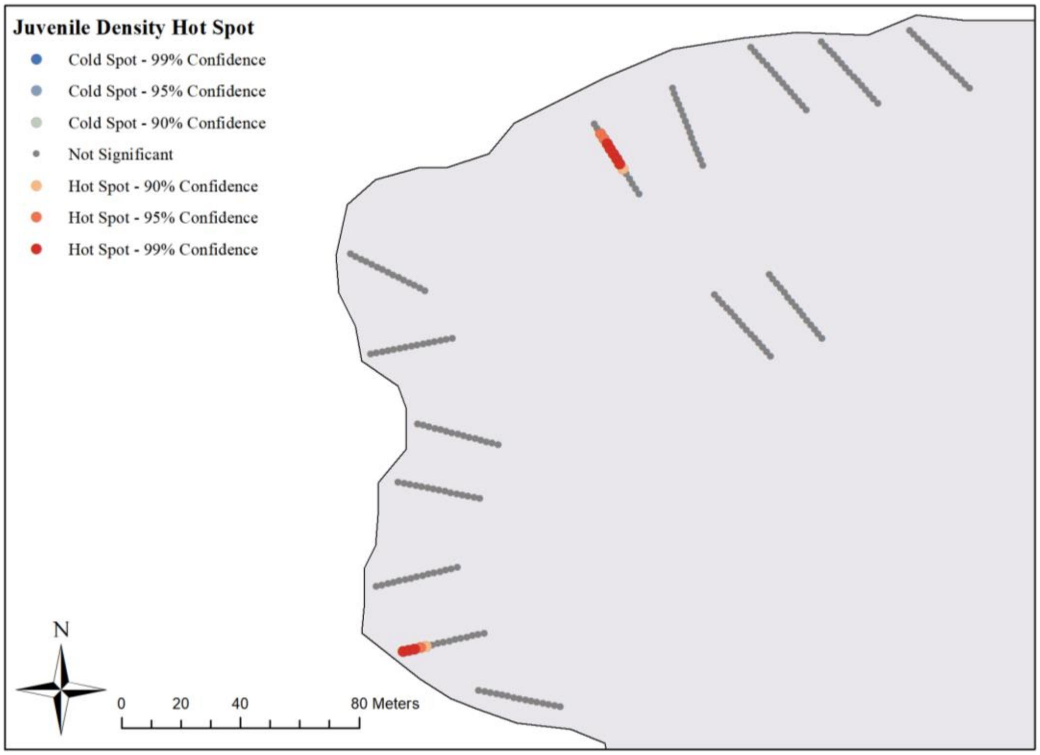
Juvenile Density Hot Spot
Cold Spot - 99% Confidence
Cold Spot - 95% Confidence
Cold Spot - 90% Confidence
Not Significant
Hot Spot - 90% Confidence
Hot Spot - 95% Confidence
Hot Spot - 99% Confidence
N
0
20
40
80 Meters
